# Comparative Microbial Modules Resource: Generation and Visualization of Multi-species Biclusters

**DOI:** 10.1371/journal.pcbi.1002228

**Published:** 2011-12-01

**Authors:** Thadeous Kacmarczyk, Peter Waltman, Ashley Bate, Patrick Eichenberger, Richard Bonneau

**Affiliations:** 1 Center for Genomics and Systems Biology, Department of Biology, New York University, New York, New York, United States of America; 2 Computer Science Department, Courant Institute for Mathematical Sciences, New York University, New York, New York, United States of America; 3 Computational Biology Program, New York University, New York, New York, United States of America; University of Virginia, United States of America

## Abstract

The increasing abundance of large-scale, high-throughput datasets for many closely related organisms provides opportunities for comparative analysis via the simultaneous biclustering of datasets from multiple species. These analyses require a reformulation of how to organize multi-species datasets and visualize comparative genomics data analyses results. Recently, we developed a method, multi-species cMonkey, which integrates heterogeneous high-throughput datatypes from multiple species to identify conserved regulatory modules. Here we present an integrated data visualization system, built upon the Gaggle, enabling exploration of our method's results (available at http://meatwad.bio.nyu.edu/cmmr.html). The system can also be used to explore other comparative genomics datasets and outputs from other data analysis procedures – results from other multiple-species clustering programs or from independent clustering of different single-species datasets. We provide an example use of our system for two bacteria, *Escherichia coli* and *Salmonella* Typhimurium. We illustrate the use of our system by exploring conserved biclusters involved in nitrogen metabolism, uncovering a putative function for *yjjI*, a currently uncharacterized gene that we predict to be involved in nitrogen assimilation.

## Author Summary

Advancing high-throughput experimental technologies are providing access to genome-wide measurements for multiple related species on multiple information levels (e.g. mRNA, protein, interactions, functional assays, etc.). We present a biclustering algorithm and an associated visualization system for generating and exploring regulatory modules derived from analysis of integrated multi-species genomics datasets. We use multi-species-cMonkey, an algorithm of our own construction that can integrate diverse systems-biology datatypes from multiple species to form biclusters, or condition-dependent regulatory modules, that are conserved across both the multiple species analyzed and biclusters that are specific to subsets of the processed species. Our resource is an integrated web and java based system that allows biologists to explore both conserved and species-specific biclusters in the context of the data, associated networks for both species, and existing annotations for both species. Our focus in this work is on the use of the integrated system with examples drawn from exploring modules associated with nitrogen metabolism in two Gram-negative bacteria, *E. coli* and *S.* Typhimurium.

## Introduction

It is now routine to have genomics data for multiple organisms of interest. For example, data may be available for both an organism of primary relevance to a specific study, as well as data for related species. Tools and algorithms for comparative analysis of multi-species datasets are therefore in high demand. Comparative analysis of gene sequences is a mainstay in computational biology [1], but comparative methods for genomics and transcriptomics data analysis are relatively new, primarily due to the fact that only recently have researchers had access to large-scale datasets from multiple species [2], [3], [4], [5], [6], [7].

A number of tools are being developed for interpreting and exploring large-scale biological networks, such as: PathSys [8], NAViGaTOR [9], BIOZON [10], [11], BN++ [12], ONDEX [13], Cytoscape, and Osprey [14]. For a review of visualization tools for systems biology see [15]. Most tools focus on automated methods for integrating interaction datasets and displaying them graphically as network diagrams. Some contain novel data structures and data models, connections to databases, and many can incorporate additional data such as, abundance, sequence, literature derived and text mining derived data. These tools often contain functions for highlighting differences in the collected datasets. While the CMMR overlaps and encompasses many of the functionalities of these previously described tools, for example visualizing network graphs from a collected data compendium, its primary function is displaying the results of multiple-species integrated biclustering analysis.

Several recent studies have shown that comparative genomics analysis improves our ability to learn regulatory interactions, co-regulated groups, and to delineate the conserved components of fundamental pathways and modules [2], [16], [17], [18], [19], [20]. In particular, multiple-species clustering and biclustering can be used to detect conserved co-regulated gene groups and serve as a foundation to begin characterizing key differences in the regulatory programs of related species. In this work we present a data visualization system that enables the visualization and exploration of integrative multi-species biclustering analysis [20]. Our interface is built on a loosely coupled system architecture that connects multiple tools and databases using the Gaggle [21], Sungear [22], and Cytoscape [23]. This interface provides coordinated access to multiple-species clusters, biclusters and networks derived from comparative genomics analysis tools such as multi-species cMonkey (MScM) [20].

### The challenges of visualizing multiple species data

The analysis of multiple species datasets presents several challenges not encountered when analyzing single species datasets. In addition to the display and exploration of multiple datatypes, such as interaction networks, cis-regulatory sequences, transcriptome and proteome data, we add the challenge of tracking connections between orthologous groups of genes. In this work we focus on exploring sets of multi-species biclusters generated with MScM. A typical multi-species biclustering (set of biclusters) will consist of:

1. The source data used to:
  - Compute the biclustering. For each species, its protein association networks, upstream sequences and expression data
  - Perform post-analytic evaluations, such as enrichment of ontology terms, i.e. GO functions and KEGG pathways
2. A set of conserved biclusters. Biclusters composed of pairs of orthologous genes spanning both species
3. Species-specific elaborations of the conserved biclusters. Following the initial generation of the conserved core of the biclusters, genes added to conserved biclusters based on evidence in a single species – including genes lacking putative orthologs in the other species
4. Species-specific biclusters. Biclusters composed entirely of genes lacking detectable orthology relationships between the two species

Our system to navigate this analysis enables exploration of both conserved biclusters, in the context of both species, and species specific additions to conserved biclusters, in the context of each individual species dataset, and illustrates general strategies for building loosely coupled systems for exploring other multi-species genomics analysis.

### Data integration across multiple species

High-throughput data exists for many microbial organisms on multiple information levels (i.e. genome sequences, transcriptomics, proteomics, metabolomics, networks of pathways and interactions). Collecting and integrating diverse and heterogeneous datasets from disparate databases is not trivial and poses a number of barriers to automating the process. One of the most significant barriers to automation of data-import is the inconsistency among the naming schemes for loci, mRNA and protein products that are employed by the major public repositories such as NCBI, Uniprot and EMBL. Versioning can also be an issue if a given data source is delayed in updating their annotations. Our resource integrates diverse data from microarray experiments, genomic sequences, and various functional associations. It utilizes a database for translating gene names across datatypes and disparate resources and ortholog names across species, and is linked to the Gaggle. We will focus our examples on two closely related γ-Proteobacteria: *E. coli* and *S.* Typhimurium.

### Multi-species integrated biclustering

Clustering and biclustering are typically used to identify groups of co-expressed genes that, ideally, represent true regulatory modules and co-functional groups such as pathways and complexes. Biclustering groups genes into condition-specific gene clusters, and can allow genes to participate in more than one bicluster. Many biclustering methods have been previously described, for example, SAMBA [24], QUBIC [25], ISA [26], BIMAX [27], and NNN [28], and other algorithms [4], [29], [30], [31], [32]. Recent integrative biclustering methods, such as MATISSE [33], the recent version of SAMBA [19], and cMonkey [18], [20] have shown that incorporating additional datatypes, such as protein interactions and cis-acting regulatory sites, improves the performance of identifying of co-functional putative co-regulated modules. There are many benefits to comparing elements among species considering that a high fraction of co-regulated modules are conserved, in whole or in part, across species [3], [34]. Recent access to multiple genomics datasets from multiple species has allowed for new comparative analyses of genomics data, for example discovering regulatory elements [35] and the MScM algorithm [20] used here. MScM learns coregulated modules by integrating expression data across subsets of experimental conditions, co-occurrence of putative cis-acting regulatory motifs in the regulatory regions of bicluster members, functional associations and physical interactions. The output consists of condition dependent conserved modules of orthologous gene groups as well as species-specific elaborations of these conserved groups. The method is a true biclustering method: a typical conserved bicluster is typically supported by a subset of the input data for each species.

### Component tools used by our system

**Figure 1:**
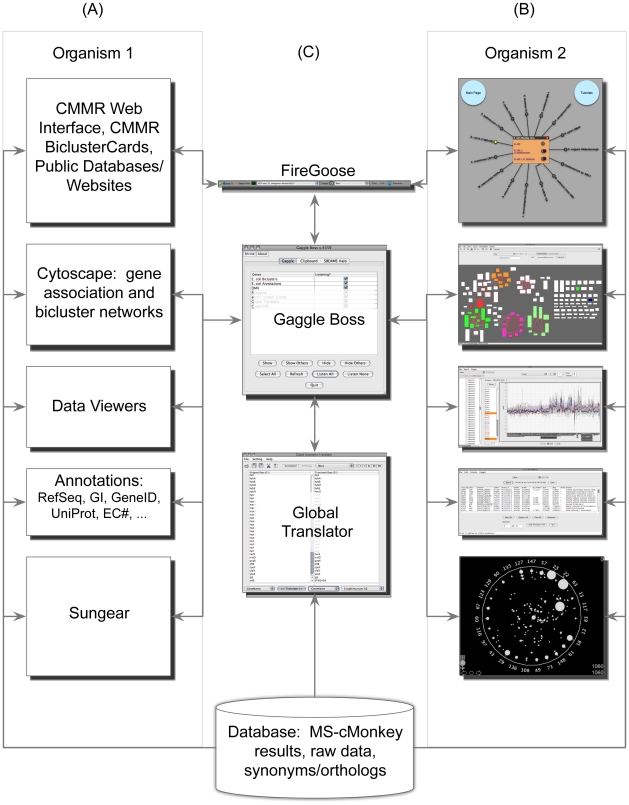
Overview of the Comparative Microbial Module Resource components (CMMR). The CMMR consists of an integrated suite of web components for visualizing the diverse aspects of the multi-species, multi-datatype analysis; facilitating access to each organism's dataset. (A) Written descriptions of the individual components for hypothetical Organism 1. (B) The corresponding graphics of each component goose displaying example data, for hypothetical Organism 2. Each of the components fetches information from the data compendium (MScM results, and raw data). (C) The CMMR integrative components: the FireGoose allows transfer of data between web pages and gaggled software, the Gaggle Boss acts as a hub for passing communications among the geese, and the Global Synonym/Ortholog Translator converts among gene annotations, accessions and translates orthologous genes between organisms. The arrows represent information flow between tools, primarily as broadcasts between tools and the Gaggle boss.

To enable exploration of a multi-species integrative biclustering result, we have constructed a system using the Gaggle and MScM (Figure 1). The Gaggle is a Java program that integrates tools by broadcasting gene, network and data selections between tools. For example, nodes selected in Cytoscape are sent to the Gaggle, which then sends the selections to all tools, which then automatically mirror those selections. The Gaggle has been shown to enable efficient creation of multi-tool systems to explore complex datasets and associated analysis [36]. Also, the loosely coupled visualization systems the Gaggle enables have several advantages including: systems-performance advantages – one tool crashing does not disable the whole system, development advantages – existing tools need not be reengineered and can be incorporated with small development costs, and maintenance advantages – due to the modularity of the resulting systems. We have extended the gaggle tools and built a corresponding database to give the user the ability to mirror gene selections in tools populated with results for one organism with the corresponding selection of the correct orthologs in the network, data, and bicluster views of another organism. Several component tools and databases are compatible with the Gaggle, or have been made compatible as part of this work, including: Sungear, Cytoscape, Cytoscape plugins such as BioNetBuilder [37], a Global Synonym/Ortholog Translator, and several tools designed to enable exploration of the genomics data available for each species (e.g. the data matrix viewer (DMV) and annotations viewer). Interactions among the CMMR, Gaggle tools and several online public databases containing annotations and genomic sequence is accomplished via a FireFox browser plugin called, the FireGoose [38] (available: http://gaggle.systemsbiology.net/docs/geese/firegoose). Selections in any tool are sent to the Gaggle which broadcasts both those gene selections to all tools for the organism in which the original selection was made and the orthologs in the other species of the selected genes. We show that this simple strategy enables effective exploration of this multi-datatype, multi-species integrative analysis.

## Materials and Methods

We present an overview of the MScM algorithm, and the system we have constructed for visualizing the resulting multiple-species biclusters. Further methodological detail, additional validation of our method, and a full description of the dataset used to demonstrate our resource can be found in the supplemental section (Text S1).

### Datasets acquisition, integration and import to our system

Microarray data was acquired from several large, public repositories such as the Gene Expression Omnibus (GEO) [39], [40], ArrayExpress [41], [42], Stanford Microarray Database (SMD) [43], [44], Many Microbes Microarray database (M3D) [45], and KEGG Expression [46], with newer datasets manually obtained from individual publications. Genomic sequences corresponding to the upstream promoter regions of each predicted gene in each genome were retrieved from Regulatory Sequence Analysis Tools (RSAT) [47], [48]. Lastly, functional associations, in the form of interaction networks, were automatically acquired from multiple sources including Prolinks [49], Predictome [50], STRING [51], [52], and MicrobesOnline [53]. We have created a data compendium containing all publicly available data for a number of microbial species including several Gram negative species *Escherichia coli*, *Salmonella* Typhimurium, *Vibrio cholerae*, *Helicobacter pylori*, *Desulfovibrio vulgaris*; three related Gram positive species *Bacillus subtilis*, *Bacillus anthracis*, *Listeria monocytogenes*, and the archeon *Halobacterium salinarum*; within this compendium all name translations have been curated to minimize error due to incorrect translation of gene synonyms. In selecting this group of microbial species, we decided to start with the two most extensively studied bacterial model organisms, *E. coli* and *B. subtilis*, and included several closely related species and some representatives from important clades of the microbial tree of life. Additional species will be included in future versions of the database, as a sufficient amount of large-scale data becomes available for those species. A full listing of all datasets used in this study for both species, including references to papers describing both original collection and several databases that aided the import and curation of the datasets, are provided in the supplemental materials (Text S1).

### Multi-species cMonkey

The MScM algorithm consists of four main steps. Beginning with step 1, putative orthologous relationships between genes in each species are identified using InParanoid [54]. InParanoid identifies not only single gene pair relationships (one-to-one) but also families of homologous genes (one-to-many, many-to-many). This allows for flexibility when considering which orthologous gene pairs to cluster. Genes are often in several putative orthology relationships and selection of orthologous pairs, one pair per putative orthology relationship, is driven by the genomics data (see Text S1 for details). After defining the set of gene pairs spanning the two species, or orthologous core, step 2 identifies the conserved biclusters via an iterative Monte Carlo optimization of the MScM score. To determine the likelihood of an orthologous gene pair belonging to a bicluster, we first simultaneously compute single-species cMonkey scores for each gene supported by each organism's individual data space (expression, common sequence motif, and connected subnetwork). Then, we compute a single, multi-species score based on the combined single-species scores. The putative-orthology based gene coupling between species is removed in step 3, where each detected conserved bicluster is split into its two constituent single-species biclusters, then species-specific additions are made separately for each species using the single-species cMonkey score. The conserved core of the bicluster detected in step 2 is kept static while species-specific additions, including both non-orthologous and orthologous genes, to the conserved biclusters are discovered via this iterative optimization. An optional step 4, not carried out in this study, identifies purely species-specific biclusters for each organism using the original cMonkey algorithm applied to genes not yet in any conserved bicluster.

We have made the cMonkey and MScM code available including tools for automating many of the data acquisition and processing steps required for assembling an integrated dataset [55]. These tools facilitate automatic queries to online biological databases for association and upstream data, such as BioNetBuilder, MicrobesOnline [53], Prolinks [49], STRING [51], [52] and RSAT [47], [48]. All input and output are stored in a MySQL database to facilitate use of the integrated dataset and MScM results by other tools. We also include a manual mode with example inputs for the algorithm both as flat files and as R data objects for those wishing to use data not in public databases. These key changes to how data is imported and stored in the MScM database and the core data-object for cMonkey and MScM are critical novel changes to the code that are required for multi-species integration and scaling of the code to much larger datasets and organisms.

### Visualizing multi-species clustering and biclusters

We created a database containing the MScM biclustering analysis data compendium for a number of microbial species. Our pipeline begins with several post-processing steps to convert cMonkey output to Gaggle compatible formats. Enrichment of functional annotations within biclusters is determined for each bicluster and the bicluster is assigned any significant annotations (p-values<0.05). A score is computed from the statistical components of each bicluster (e.g. residual, functional enrichment significance values). Specifically, the bicluster score is computed using Stouffer's z-score method for meta-analysis from a collection of bicluster statistics. Data files are generated for the complete bicluster network and the subnetwork of related biclusters before the website for a result is generated. Lists of orthologous genes between each species are generated as part of the analysis and loaded into the synonym/ortholog database.

### Multi-species extension of the Gaggle

To mirror selections simultaneously in several tools that visualize different aspects of the data, the results and the comparison between species we utilize the Gaggle, a loosely coupled system of web applications (geese) [21]. The Gaggle is a software framework that integrates independent application tools and biological data into an environment that allows the exchange of data among tools. All of the tools employed in our resource are Java web-starts or directly integrated into the web interface, thus removing any barrier to use based on tool compatibility, installation or data-transfer. The Gaggle also serves to coordinate the deployment and interoperation of these Java Web Start tools. Each individual application, or goose, can be launched with the click of a button on the BiclusterCard. The geese included in the resource are: a Global Synonym/Ortholog Translator, BioNetBuilder (Cytoscape plug-in), the FireGoose, Data Matrix Viewer, Annotations viewer, Cytoscape – bicluster network and gene network viewers, and Sungear. All the tools are connected through a communication hub called the Gaggle Boss, which passes simple messages among the geese, called broadcasting, summarized in Figure 1. When a broadcast is received, the goose will display the relevant information for the data. BiclusterCards and online databases (e.g. STRING, KEGG, etc.) connect to the tools through the FireGoose, a browser plug-in for Firefox adding the capability to communicate with the Gaggle. Embedded in each BiclusterCard is microformat code containing metadata for properties such as gene names, bicluster nodes, and condition names that can be broadcasted to other geese. The Bicluster Network viewer is a Cytoscape goose that displays a network of bicluster interactions, where nodes are biclusters, and edges are any shared properties (e.g. functional annotation, gene overlap, etc). Similarly the Gene Associations viewer is a Cytoscape goose that displays the gene associations from the data compendium. A Data Matrix Viewer goose acts as a spreadsheet program that can display and plot gene expression values. The Annotations goose displays a table of the genes and their various annotations specific to a single organism, for example, locus tag, gene name, protein id, and gene id accession. There is a Global Synonyn/Ortholog Translator that, given a list of genes from one species, can display the orthologous genes from another species. Lastly, the MScM output showing gene expression, gene subnetwork, sequence motifs, and motif locations in promoter sequence, can be displayed in the ClusterInfo Viewer.

### The web and Gaggle interface to our multi-species biclustering

**Figure 2:**
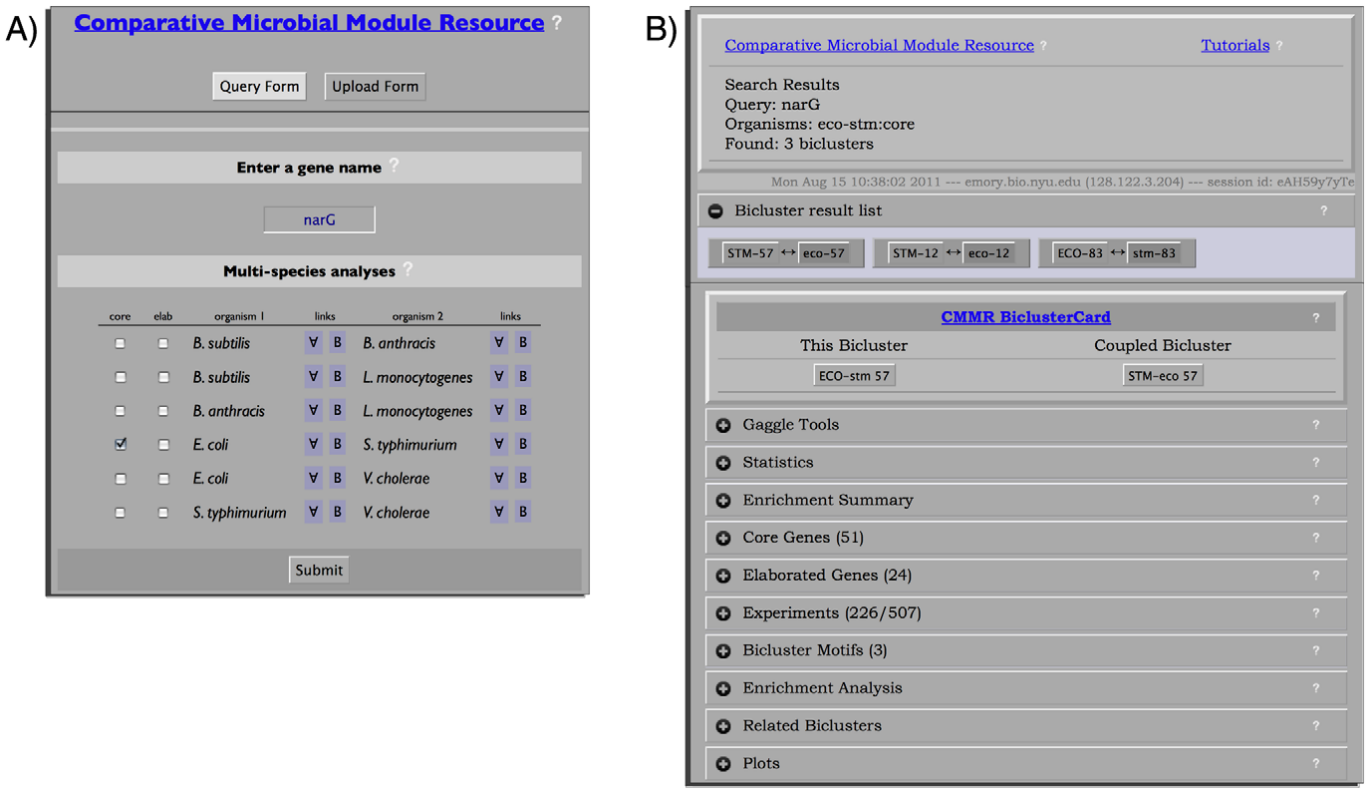
CMMR Query Page and BiclusterCard. The CMMR web interface allows users to search for biclusters of interest, with each resulting bicluster displayed in a BiclusterCard format. (A) The CMMR search page showing the title links to the CMMR wiki, query form button, upload form button, and input fields. Shown is the query form with an example search for *narG* in the core set (check box) of bicluster gene members for a MScM run of *E. coli* – *S.* Typhimurium. (B) The result page from this search – a user has access to the CMMR wiki, tutorials, a brief description of the search query, the resulting bicluster list and BiclusterCards. The BiclusterCard contains links to Gaggle tools, and expandable/collapsible tabs to display the bicluster's diverse supporting information. There are help icons with mouseover tooltips for descriptions and information.

A web interface was implemented to facilitate exploration of the multi-species biclusters. The starting page allows users to create several types of queries and contains a text box to input a gene name or group of genes, select boxes to choose bicluster sets from single and, core or elaborated MScM analyses, and a submit button to begin the search for biclusters containing the gene or genes of interest from the selected biclustering analyses (Figure 2A). Any biclusters returned from a search are presented as a list ranked by bicluster score. A first step in organizing the diverse information contained in, and supporting each bicluster was to create a system for generating bicluster summaries that link to online tools and source data. To this end, for each bicluster, our system creates a ‘BiclusterCard’. Each BiclusterCard provides the following information in the form of expandable/collapsible tabs (Figure 2B):

- Gaggle tools: Embedded links to integrated software tools
- Statistics: The number of genes and conditions in the bicluster, score, residual, mean motifs p-value, motif E-values
- Enrichment Summary: based on the most significant annotations from COG, KEGG and GO enrichment analysis
- Core Genes: Genes table for conserved core members of the bicluster– including GO, KEGG, and COG gene annotations
- Elaborated Genes: Same as above, but for elaborated members of the bicluster
- Experiments: Table with links to the meta-data and primary articles
- Bicluster Motifs: if any motifs were found, the sequence logo is displayed here along with matches to any known motifs
- Enrichment Analysis: Tables for GO, KEGG, and COG annotation enrichment – with description and significance values
- Related Biclusters: Table with links to biclusters with similar functional/pathway annotations, similar motifs, or overlapping gene members
- Plots: Bicluster plots for gene expression profiles, mean gene expression, and expression heatmap

Each element of the bicluster card is generated automatically by our system, is compatible with outputs from other widely used biclustering tools, and provides links to descriptions/tutorials for using the linked tools or databases.

## Results/Discussion

Visualizing the entire multiple-species dataset and integrative biclustering analysis at once, in a single view or tool, is cumbersome and ineffective at conveying biologically useful information due to the scale and multitude of different relationships in the data and analysis. Therefore, a main goal of our resource is to design an interface that provides access to the MScM results and collected data compendium via multiple queries (e.g. query by pathway, gene, network neighborhood, bicluster or ontology term). Although multiple queries are possible it is envisioned that a user will typically begin by querying for a gene or group of genes and browse MScM gene modules. A user can then begin exploring relationships between datasets for individual genes, subnetworks of genes, among modules, or among modules with particular shared attributes, such as, functional annotation. The system also allows high-level manipulation of queries, i.e. queries and operation on results of past queries, via Sungear. Examining the intersections, complements, and unions of module gene memberships, or identifying common promoter elements among genes in a module or among modules can be performed using Sungear following several broadcasts of gene lists. Gene lists are typically the results of queries, neighbors in a network loaded into the cytoscape goose, or the members of biclusters. These are just few examples of how a user can use the resource. Moreover, all of this functionality is automatically performed (mirrored) across species multiple species datasets.

To demonstrate our resource's capabilities, we explore nitrogen metabolism associated multi-species biclusters with the specific biological goal of identifying new genes functionally associated with nitrogen metabolism in *E. coli* and *S.* Typhimurium. For a global validation of our multi-species biclustering method and a detailed comparison of our method to several other methods, as well as a detailed description of the complete dataset used in this study see the supplemental section (Text S1) provided in the electronic version of this article. The CMMR is available at http://meatwad.bio.nyu.edu/cmmr.html.

### Exploring nitrogen metabolism in an *E. coli* and *S.* Typhimurium integrated genomics dataset

Nitrogen is an essential input into several metabolic pathways including amino acid and nucleotide biosynthesis, and can act as a terminal electron acceptor in dissimilatory nitrate reactions [56]. It is common for some microbes including *E. coli* to use nitrogen for energy-harvesting purposes in anaerobic and nutrient depleted conditions [56]. A central component of nitrogen assimilation and metabolism is nitrate reductase, a membrane bound enzyme that catalyzes the conversion of nitrate to nitrite. The *narGHJI* operon encodes the multiple subunits of nitrate reductase A in *E. coli*. The following section sequentially guides the reader through using our system to explore biclusters containing genes in the *nar* operon and other nitrogen metabolism associated genes. A web tutorial for the use of our system can also be found at: http://meatwad.bio.nyu.edu/psbr/index.php/Tutorials

### Identifying a potential role for unknown genes in biclusters containing nar genes

**Figure 3:**
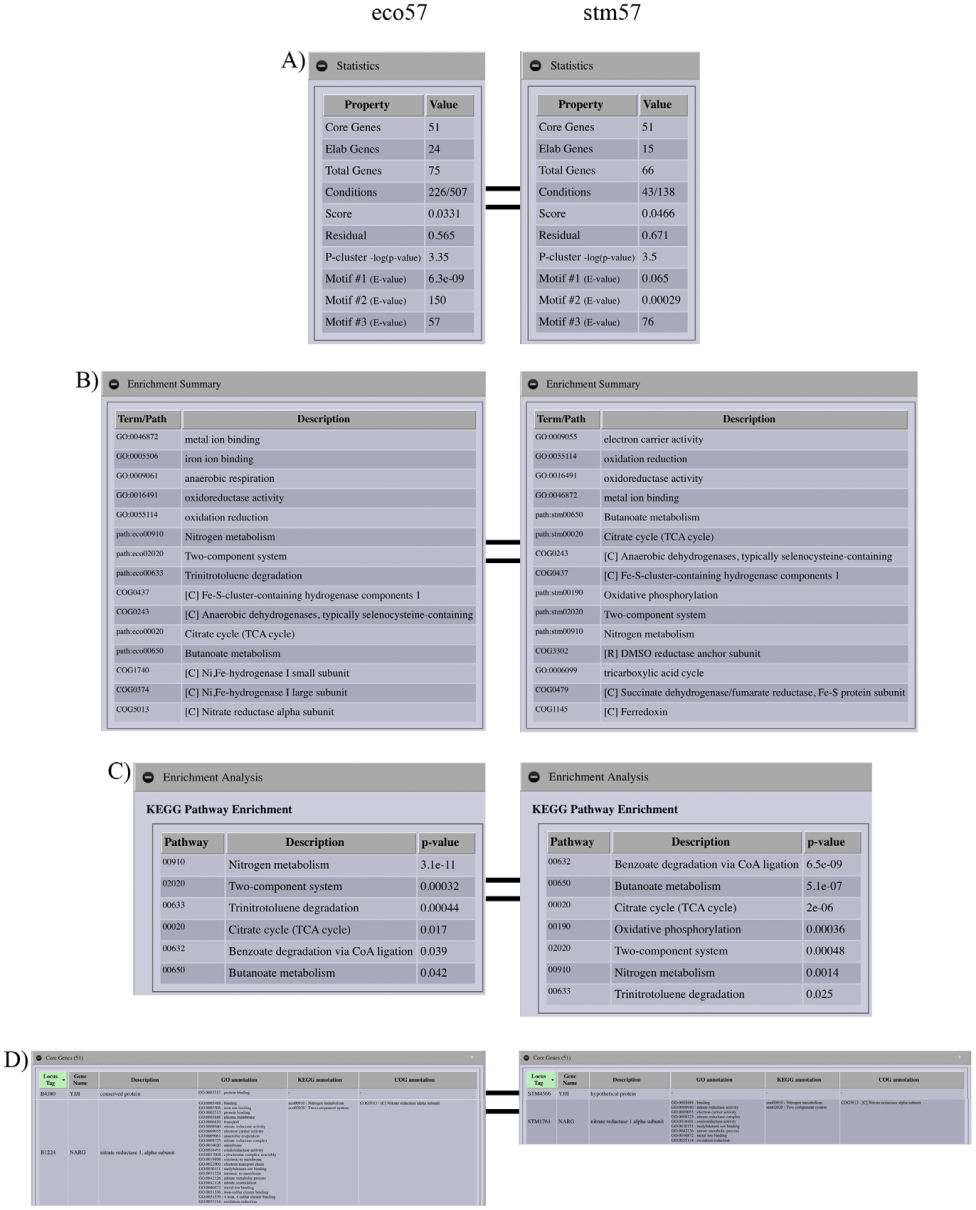
BiclusterCard components I: Statistics, Enrichment Summary, Core Gene Table, KEGG Pathway Enrichment. The BiclusterCard is a summary of the information supporting a bicluster, including links to online tools and source data. Shown in the figure are the expanded tabs for: statistics, enrichment summary from COG, GO and KEGG enrichment analysis, KEGG pathway enrichment, and core gene table for multi-species bicluster *E. coli* – *S.* Typhimurium bicluster 57. (A) Statistics tab for eco57 (left) and stm57 (right) displays a table with the following columns: Property and Value. The information contained in this table includes: the number of core and elaborated genes, fraction of conditions in the bicluster, the bicluster score, bicluster residual, bicluster mean p-value (mean of all motifs found in the promoter sequences), and the E-value for each motif found in the bicluster. (B) Enrichment Summary tab for eco57 (top) and stm57 (bottom) displays a table with the following columns: Term/Pathway and Description. This table lists the most significant annotations from ontological enrichment tests of COG, KEGG pathway, and GO annotations. (C) The Functional Enrichment tab displays tables listing the significant annotations from the COG, GO and KEGG enrichment analyses. Shown is the KEGG pathway enrichment table for eco57 (top) and stm57 (bottom). The table consists of the following columns: Pathway, Description, and p-value. Each column can be sorted. (D) Core Gene tab for eco57 (top) and stm57 (bottom), showing the number of core genes (51), and a table containing the following columns: Locus Tag, Gene Name, Description, GO annotations, KEGG annotations, and COG annotations. Locus Tag, Gene Name and Description columns can be sorted.

We begin our exploration of identifying conserved biclusters containing *nar* genes by searching for “narG” in the core set of genes from an *E. coli* and *S.* Typhimurium MScM bicluster set (Figure 2A). Explicitly, typing ‘narG’ into the gene-name textbox, selecting the core checkbox and clicking ‘submit’ on the CMMR start page, will retrieve any biclusters containing *narG* in the core set of genes. The results page returned following our “narG” query includes a header with links to the CMMR wiki, links to tutorials, a description of the search query and a list of any retrieved biclusters, in this case 3 biclusters were found (Figure 2B). There is a button for each bicluster that will display its BiclusterCard (see materials and methods). Looking at the first BiclusterCard for *E. coli* bicluster-57 (eco57), we will click on the ‘Coupled Bicluster’ button to open the BiclusterCard for *S.* Typhimurium bicluster 57 (stm57). Expanding the ‘Statistics’ tab shows that eco57 contains 75 genes (51 core genes, 24 elaborated genes), 226 experiments, whereas stm57 contains 66 genes (51 core genes, 15 elaborated genes) and 43 experiments (Figure 3A). This first table highlights differences in gene membership of the two biclusters. The ‘Enrichment Summary’ shows similar but not identical annotations involved in various metabolic activities related to anaerobic respiration and energy production from nitrogen for both biclusters (Figure 3B). The ‘Experiments’ tab shows that expression of these genes changes under a variety of conditions including: stress, growth on minimal media, anaerobic metabolism, and DNA damage. Expanding the ‘Enrichment Analysis’ tab displays tables containing significant COG, GO and KEGG annotations. We can see that eco57 and stm57 differ in the ranking of the KEGG pathway annotations and stm57 includes an additional pathway (Figure 3C). This could reflect slightly different uses of these modules in these organisms or discrepancies in the gene annotations.

**Figure 4:**
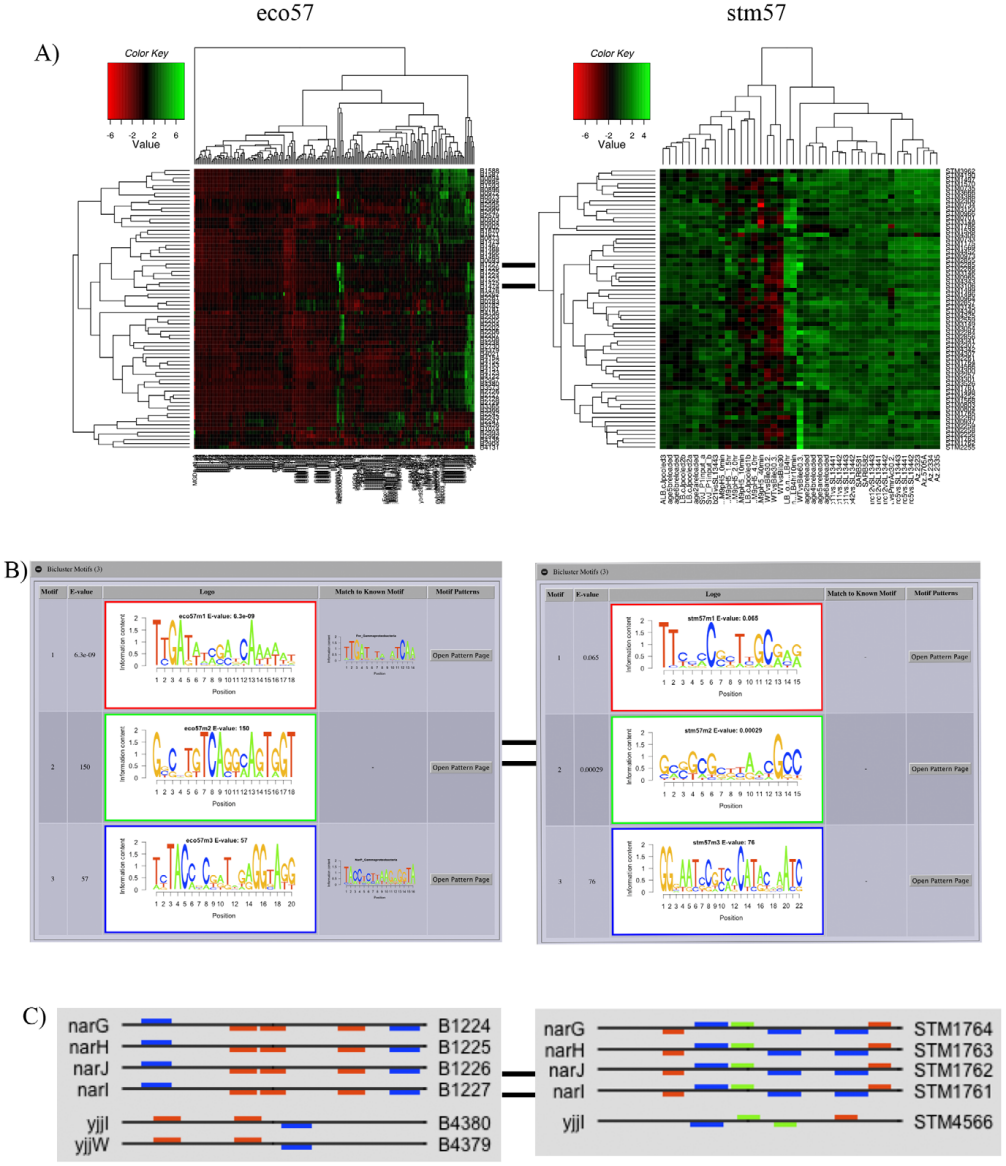
BiclusterCard components II: Bicluster Motifs, Upstream Patterns, Plots. Shown in the figure are, the expanded tab for Plots displaying a gene expression heatmap, the expanded tab for Bicluster Motifs, and an example of the upstream motif patterns for multi-species bicluster *E. coli* – *S.* Typhimurium bicluster 57. (A) Example plot of a gene expression heatmap for the bicluster genes and conditions in eco57 (left) and stm57 (right); upregulated expression (green) and downregulated expression (red). (B) Putative regulatory sequence motifs found in bicluster gene member promoters for eco57 (left) and stm57 (right). The table displays a row for each motif found and columns for the motif number, E-value, sequence logo, matches to any known motifs, and a link to motif pattern page. Eco57 motif #1 matches the known FNR binding sequence and motif #3 matches the known NarP binding sequence. (C) The promoter motif patterns for the motifs shown in (B) for eco57 (left) and stm57 (right). The location of the motifs are represented by colored rectangles on the promoter sequence (black line) and the colors correspond to the logo border colors seen in (B); motif #1 (red), motif #2 (green) motif #3 (blue). For the bicluster gene members shown, bicluster motifs #1 and #3 appear in the promoter regions of the eco57 members, whereas all three bicluster motifs appear in the promoters for the stm57 members. The identical motif pattern indicates MScM has determined them to be in an operon. It is known that *narGHJI* exist as an operon, but MScM has determined that *yjjI* is in an operon with *yjjW* (this is also predicted by [62]). However, *yjjW* is found only in the elaborated gene set of eco57 and it is not found in stm57.

Then, looking at the gene GO, KEGG and COG annotations by expanding the ‘Core Genes’ tab we see many genes have the same or similar annotations and some have either none or different annotations such as *narG* and *yjjI* (Figure 3D). Finally, under the ‘Plots’ tab we can view plots for gene expression profiles, bicluster mean expression, and an expression heatmap – to visualize differences in clustering bicluster gene members (Figure 4A).

Expanding the ‘Bicluster Motifs’ tab displays the motifs detected in the bicluster. Two of the detected motifs for eco57 show similarity to known nitrate/nitrite response transcriptional regulator binding motifs (Figure 4B). Motif #1 matches the *E. coli* FNR (fumarate and nitrate reduction) binding consensus sequence (TTGAT N4 ATCAA) [57] and eco57 motif #3 corresponds to the NarP binding sequence [58], [59]. The sequence motifs of stm57 show no notable similarity to known motifs. The FNR homolog in *S.* Typhimurium, *oxaR*, has a similar but less defined consensus sequence [60], which could account for the lack of association with stm57 motif #1. The promoter motif patterns display which gene members share common motifs and the location in the gene's upstream sequence. Identical motif patterns indicate they are an operon, such as operon *narGHJI* (Figure 4C). MScM and MicrobesOnline [61], [62] predict *yjjI* and *yjjW* to be in an operon, which is reflected in eco57 (*yjjW* is present in the elaborated gene set) but not stm57 (Figure 4C). Exploring the correspondence of the MScM detected motifs with known nitrogen metabolism motifs increases our level of confidence that this bicluster is truly coregulated in both organisms.

**Figure 5:**
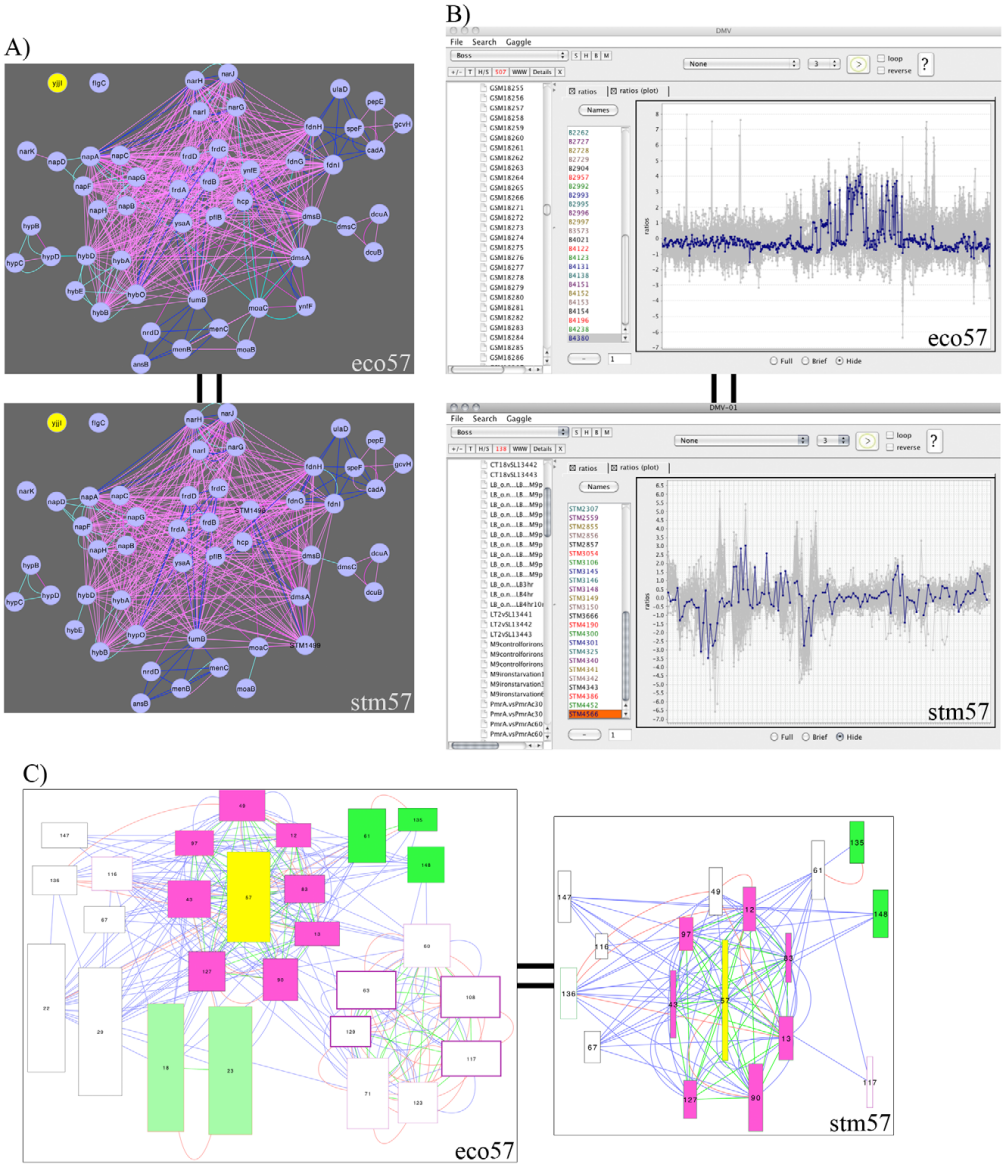
CMMR linked Gaggled tools I: Gene Network, Data Matrix Viewer, Bicluster Network. Expanding the Gaggle tools tab on the BiclusterCard for multi-species bicluster *E. coli* – *S.* Typhimurium bicluster 57, reveals a list of links (buttons) to the various Gaggle tools. (A) The Gene Associations button opens a Cytoscape goose that displays the core genes subnetwork for eco57 (top) and stm57 (bottom). The nodes represent genes and edges represent associations based on data from the compendium, indicated in yellow is gene *yjjI*. Edges are shared annotations: COG code (pink), Prolinks phylogenetic profile (purple), metabolic pathway (blue), operon (light cyan), and Predictome phylogenetic pattern (dark cyan). (B) The expression profiles for the genes and conditions from eco57 (top) and stm57 (bottom) can be explored by opening the Data Matrix Viewer. Using the FireGoose, the bicluster's genes and conditions can be broadcast from the BiclusterCard. We can see how the expression profile of gene *yjjI* (indicated by the colored line) matches other profiles in the bicluster. (C) The Bicluster Network button opens a Cytoscape goose to display the complete bicluster network where each node is a bicluster (width and height proportional to number of genes and conditions, respectively) and edges represent any shared properties and annotations. We can explore the related bicluster subnetwork for bicluster 57 (yellow), eco57 (left) and stm57 (right), by broadcasting the list of related biclusters (using the FireGoose) from the BiclusterCard to select those biclusters and display them in a new window. There are 10 additional biclusters in the eco57 subnetwork. Node fill color represents significant COG annotation, border color represents significant GO annotation, node border thickness represents residual, and edge color represents shared COG (green) KEGG (red), or GO (blue) annotations.

Among the core gene list for this bicluster, *yjjI* is described only as encoding a conserved protein with no functional annotation (Figure 3D). To examine this gene in the context of multiple network-types, the original data, and the biclustering, we now open several Gaggle tools, including the bicluster and gene network Cytoscape geese, Data Matrix Viewer and BioNetBuilder. First, we explore associations between core gene members of eco57 and stm57. For the 51 genes in the core gene member subnetworks, eco57 has 518 associations and stm57 has 420 edges, with no associations for *yjjI* (Figure 5A; associations shown are operon edges, metabolic pathway edges, phylogenetic profile edges, and protein interaction edges between genes in different biclusters). Next, we explore the expression profiles of the bicluster gene members and conditions by broadcasting them to the Data Matrix Viewer. Selecting *yjjI*, we can see that it has similar expression to other bicluster gene members (Figure 5B). Thus, the data (sequence motifs, associations, expression) supports eco57 and stm57 as coherent, putatively coregulated gene groups, and gene *yjjI*, while lacking associations, is supported by common motifs and correlated expression. We can use more Gaggle tools to search for additional information characterizing the bicluster gene members, particularly *yjjI*. For example, broadcasting the gene members to BioNetBuilder, we can browse protein structure and functional predictions. YjjI is predicted to have a domain structure that matches a “Class III anaerobic ribonucleotide reductase NRDD subunit” [63] and a function prediction of oxidoreductase activity [64], [65]. If we broadcast *yjjI* to other online databases such as Entrez Gene [66], we find that *yjjI* is adjacent to *yjjW*, but no information that they are in an operon. As mentioned above, both MScM and MicrobesOnline have predicted them to be in an operon. There is further information from EcoGene [67] reporting *yjjI* as an ortholog of *H. influenzae hi0521*, which is a *pflB* homolog and coding for a formate acetyltransferase [68]. Taken together, this information suggests a role for YjjI in nitrogen metabolism. It is important to note that a corresponding single-species bicluster in *E. coli* was not found (in the *E. coli* single species cMonkey run we find no bicluster with significant gene overlap to this significant conserved bicluster), further illustrating the importance of the MScM method. However, the species-specific elaborations of the bicluster may display additional information, such as, individual adaptations to this metabolic process.

Another possible use of our system is the exploration of collections of biclusters to identify novel interactions among modules. In the context of this example we can extract the subnetwork of biclusters related to the nar bicluster described above from a network that displays associations among biclusters by broadcasting the list of biclusters related to the orthologous core from the BiclusterCard to the Bicluster Network Viewer (Figure 5C). Biclusters are nodes with width and height proportional to the number of genes and conditions, respectively, and shared significant KEGG pathway, COG function, and GO function annotations are edges. The subnetwork shows 38 related biclusters for *E. coli* and 33 biclusters for *S.* Typhimurium. In this subnetwork there are several biclusters containing gene modules highlighting complementary interactions such as: amino acid biosynthesis/metabolism pathways and glutamate metabolism (bicluster-61); NADH dehydrogenase, succinate dehydrogenase (bicluster-43), citrate fermentation (bicluster-147), and amino acid ABC-type transporters (bicluster-148). This highlights the presence of conserved core interactions among eco57 and stm57 with other modules and independent species-specific modifications within these modules.

**Figure 6:**
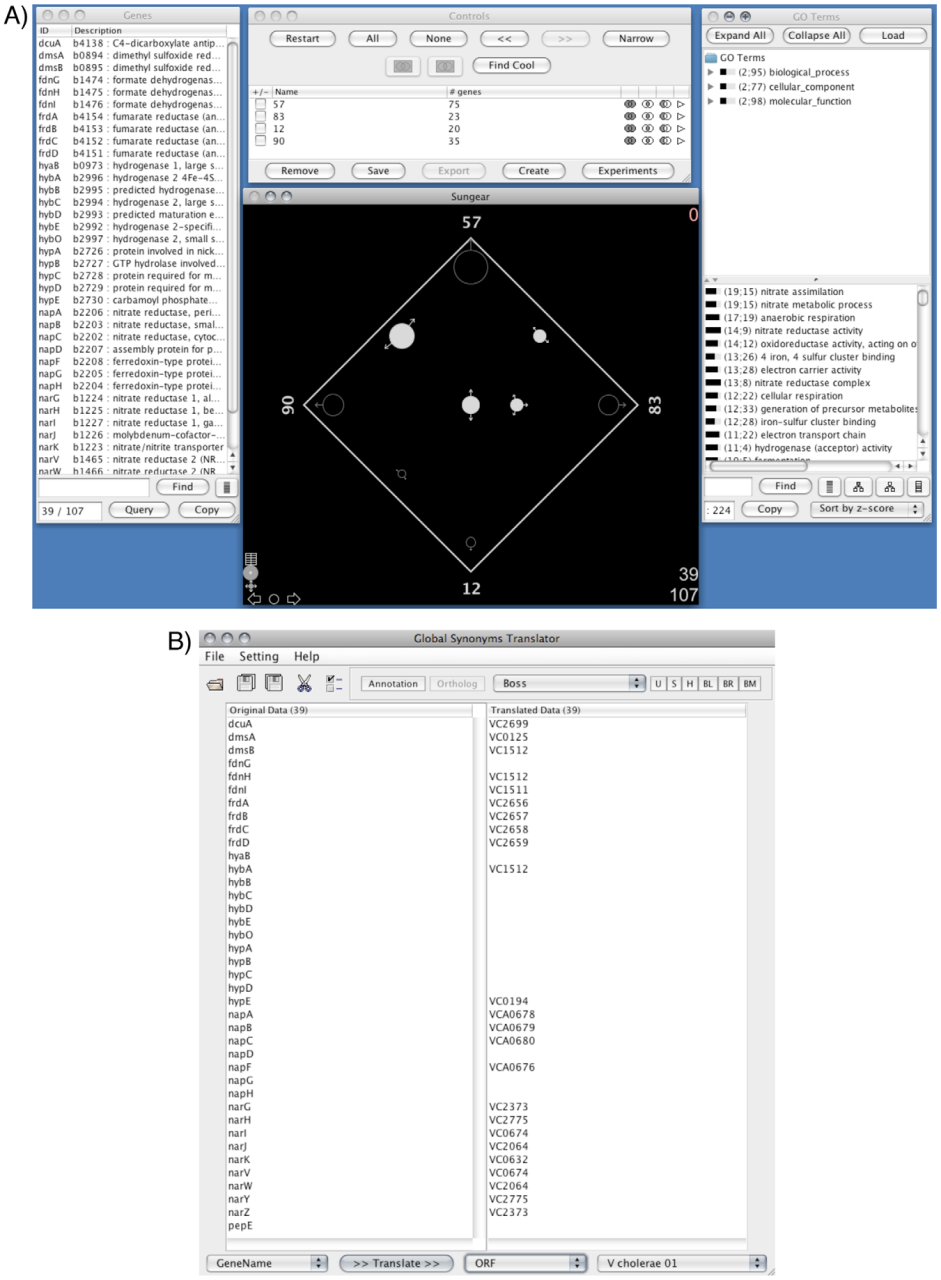
CMMR linked Gaggled tools II: Sungear and Global Synonym/Ortholog Translator. The Sungear goose is a visualization tool capable of displaying set relationships and operations (intersections, complements, unions). In this case, sets are gene lists from a gaggle broadcast. (A) Four biclusters were broadcast to Sungear: eco57, eco83, eco12, and eco90. Each bicluster is represented as a vertex or anchor on the square and the circles, called vessels, represent the intersection of elements, in this case, bicluster gene members (bottom center window). Selected are four circles (filled circles) representing the intersections of gene members for bicluster 57 with the other three biclusters, 83, 12, and 90. The list of genes from the selected sets is seen in the gene list window (left window). Manipulation of the sets is done through the control window (top center window). Over representation of GO terms are shown in the GO term window (right window). (B) The list of 39 *E. coli* genes (left panel) was broadcast to the Global Synonym/Ortholog Translator to find 24 putative orthologous genes (right panel) in *V. cholerae*.

We can further explore nitrogen metabolism in the context of *V. cholerae*. First, we launch the Sungear goose and the Global Synonym/Ortholog Translator. From the subnetwork of related biclusters we select bicluster 57 and the top 3 overlapping biclusters (based on the ‘Related Biclusters -> Core Related’ table: 12, 83, 90). We then broadcast these 4 biclusters to the Sungear goose, select all groups and create a Sungear plot (Figure 6A). Next we select the vessels that have intersections with bicluster 57, yielding 39 genes. These *E. coli* genes are then broadcast to the Global Synonym/Ortholog Translator where we obtain 24 orthologs in *V. cholerae* (Figure 6B). Now, we launch the *V. cholerae* Bicluster Network Viewer by clincking the ‘B’ button on the CMMR start page next to the *E. coli* – *V. cholerae* MScM analysis. After the network has loaded, we highlight any biclusters containing those genes by broadcasting the translated orthologs to the bicluster network. This reveals 27 biclusters, of which only 3 are enriched for genes involved in nitrogen metabolism. Further investigation of the *E. coli* – *V. cholerae* MScM analysis shows that bicluster 109, a highly significant bicluster enriched for nitrogen metabolism in *E. coli* (eco109) but not *V. cholerae* (vch109), is absent from this list. Rather, vch109 is enriched for genes involved in molybdate ion transport and sulfur metabolism. The genes involved in nitrogen metabolism in eco109 are found in the elaborated set and not in the conserved core. This could represent a possible species-specific difference between these two organisms.

Using the CMMR, much knowledge was uncovered from the search of just a single gene, *narG*. In one case, for a currently uncharacterized gene, *yjjI*, the gathering of diverse information such as: putative orthology between two species, co-expression and common putative regulatory motifs with other bicluster genes, and a prediction for the protein's structure and function, was facilitated by the various BiclusterCards and Gaggle tools.

### Conclusions

We have developed a publicly accessible web resource for comparative genomics studies of several prokaryotic organisms, with plans to expand this resource over time. As described above, in our example with coupled *E. coli* – *S.* Typhimurium bicluster 57, the combination of our method for simultaneously biclustering multiple datasets from multiple species and easy to use exploration system quickly led to novel biological insights and generate an informed hypothesis about the involvement of gene *yjjI*, a currently uncharacterized gene, in nitrogen metabolism. The complexity and richness of the results of comparative genomics data analysis requires a system like the one presented here. We present specific examples of the use of our system in the hopes of sparking discussion about what the next generations of comparative genomics analysis and visualization systems should look like. Our paper focuses on the combined, multi-tool interface required by biologists wishing to explore the biological significance and function of multi-species, multi-datatype biclusters and their species-specific elaborations and deletions. An important aspect of our system is the ability to submit new data for analysis and integrate the results into the resource for public access. We provide multiple avenues for researchers wishing to build this system for their species of interest, such as publicly available tools and code, and/or we will run our analysis and build this system for researchers without computational resources.

The CMMR wiki is intended to be a platform for information exchange, encouraging the contributions of researchers who use the resource, whether via curation or suggestions of new tools. Improvements to the resource could be made 1) in method development, for example, further optimization of the MScM algorithm and inclusion of additional analysis methods, 2) as datasets become available, increasing the number of included species, and 3) as further development and invention of intuitive visualization and exploration tools manifest. This effort could also serve as a framework for applications to comparative biclustering of eukaryotic organisms.

## Supporting Information

Text S1 **Supporting information.** The supporting information file includes descriptions of: the *E. coli* – *S.* Typhimurium dataset analyzed, pseudocode for the MScM algorithm, validation of the method's performance on the *E. coli* – *S.* Typhimurium dataset, and the highlighted biclusters.
(DOC)
