# Supplementary material for: Comparative Microbial Modules Resource: Generation and Visualization of Multi-species Biclusters
Source: PLoS Comput Biol. 2011 Dec 1;7(12):e1002228. doi: 10.1371/journal.pcbi.1002228 (PMC3228777; doi:10.1371/journal.pcbi.1002228)
Supplement: Text S1 — Supporting information. The supporting information file includes descriptions of: the E. coli – S. Typhimurium dataset analyzed, pseudocode for the MScM algorithm, validation of the method's performance on the E. coli – S. Typhimurium dataset, and the highlighted biclusters. (DOC) [file pcbi.1002228.s001.doc]

Table of Contents

Materials [2](#__RefHeading___Toc163897653)

[Dataset analyzed](#_Dataset_analyzed) [2](#__RefHeading___Toc163897654)

Methods [4](#__RefHeading___Toc163897655)

[MScM Algorithm Pseudocode Overview](#_MScM_Algorithm_Pseudocode) [4](#__RefHeading___Toc163897656)

Validation [6](#__RefHeading___Toc163897657)

[Overview of the bicluster comparison metrics](#_Overview_of_the) [6](#__RefHeading___Toc163897658)

[Quick-glance table for pairings involving *E. coli* – *S.* Typhimurium](#_Quick-glance_table_for) [7](#__RefHeading___Toc163897659)

[Additional (bi)cluster coherence metric figures](#_Additional_(bi)cluster_coherence) [9](#__RefHeading___Toc163897660)

[Residuals](#_Residuals) [9](#__RefHeading___Toc163897661)

[Average pairwise correlations](#_Average_pairwise_correlations) [10](#__RefHeading___Toc163897662)

[Network Association p-values](#_Network_Association_p-values) [11](#__RefHeading___Toc163897663)

[Additional size distribution, overlap and coverage figures](#_Additional_size_distribution,) [12](#__RefHeading___Toc163897664)

[Number of genes](#_Number_of_genes) [12](#__RefHeading___Toc163897665)

[Number of conditions](#_Number_of_conditions) [13](#__RefHeading___Toc163897666)

[Coverage (matrix element-wise)](#_Coverage_(matrix_element-wise)) [14](#__RefHeading___Toc163897667)

[Overlap (matrix element-wise)](#_Overlap_(matrix_element-wise)) [15](#__RefHeading___Toc163897668)

[GO and KEGG annotation enrichment](#_GO_and_KEGG) [16](#__RefHeading___Toc163897669)

Description of highlighted biclusters [17](#__RefHeading___Toc163897670)

[*E. coli* bicluster 57](#_E._coli_bicluster) [17](#__RefHeading___Toc163897671)

[*E. coli* bicluster 57 core gene list](#_E._coli_bicluster_1) [17](#__RefHeading___Toc163897672)

[*E. coli* bicluster 57 elaborated gene list](#_E._coli_bicluster_2) [20](#__RefHeading___Toc163897673)

[*S.* Typhimurium bicluster 57](#_S._typhimurium_bicluster) [21](#__RefHeading___Toc163897674)

[*S.* Typhimurium bicluster 57 core gene list](#_S._typhimurium_bicluster_1) [21](#__RefHeading___Toc163897675)

[*S.* Typhimurium bicluster 57 elaborated gene list](#_S._typhimurium_bicluster_2) [23](#__RefHeading___Toc163897679)

# Materials

## Dataset analyzed

The *E. coli* expression data matrix consisted of 507 conditions from 16 projects acquired from the Many Microbe Microarrays Database (M3D) [37] covering various conditions including: genetic perturbations, changes in oxygen concentration and pH, growth phases, antibiotic treatment, heat shock, and different media.

The *S.* Typhimurium expression data matrix consisted of 138 conditions from 8 studies acquired from the Stanford Microarray Database (SMD) [36] covering various conditions including: chemical effects, nutrient limitation, library verification, strain comparison, media comparisons, time course, and mutants.

**Table 1**: Total number of genes, conditions, and association edges in each dataset used for the multi-species analysis, by organism.

| Number of: | | ***E. coli*** | ***S.* Typhimurium** |
| --- | --- | --- | --- |
|  | **Genes** | 4264 | 3745 |
|  | **Conditions** | 507 | 138 |
| Association edges | | | |
| Source | Egde type |  |  |
|  | **Operon** | 3414 | 2104 |
| KEGG | **Metabolic** | 96931 | 75363 |
| Prolinks | **Gene Neighbor** | 29228 | 29942 |
| Prolinks | **Phylogenetic Profile** | 20058 | 20094 |
| Prolinks | **Gene Cluster** | 6048 | 6476 |
| COG | **COG-code** | 644856 | 379484 |

**Table 2**: Total number of orthologs, orthologous families, and ortholog pairs generated by InParanoid.

| Number of: | ***E. coli*** | ***S.* Typhimurium** |
| --- | --- | --- |
| **orthologous groups** | 2827 | |
| **orthologous pairs** | 2856 | |
| **multi-member groups** | 22 | |
| **Remaining unique genes** | 2836 | 2845 |

# Methods

## MScM Algorithm Pseudocode Overview

Define *organisms*, *orthologs*, *num.biclust*, and *iter.max* to be each organism’s dataset (expression, genomic sequence, network associations), putative orthologs between the organisms, the number of biclusters to search for, and the maximum number of iterations for the procedure, respectively. The method is a Monte Carlo optimization that, given a bicluster seed, optimizes a bicluster by iteratively adding or dropping genes and conditions according to the multi-species score (*gain*). The individual likelihoods for the *gain* for expression, sequence, and association networks, are represented by *r*, *s*, and *q*, respectively. The membership probability (*prob_membership_*) of becoming part of the bicluster is based on the *gain* and the decision boundary formed using logistic regession (model). See [12] for the complete description of the method.

**
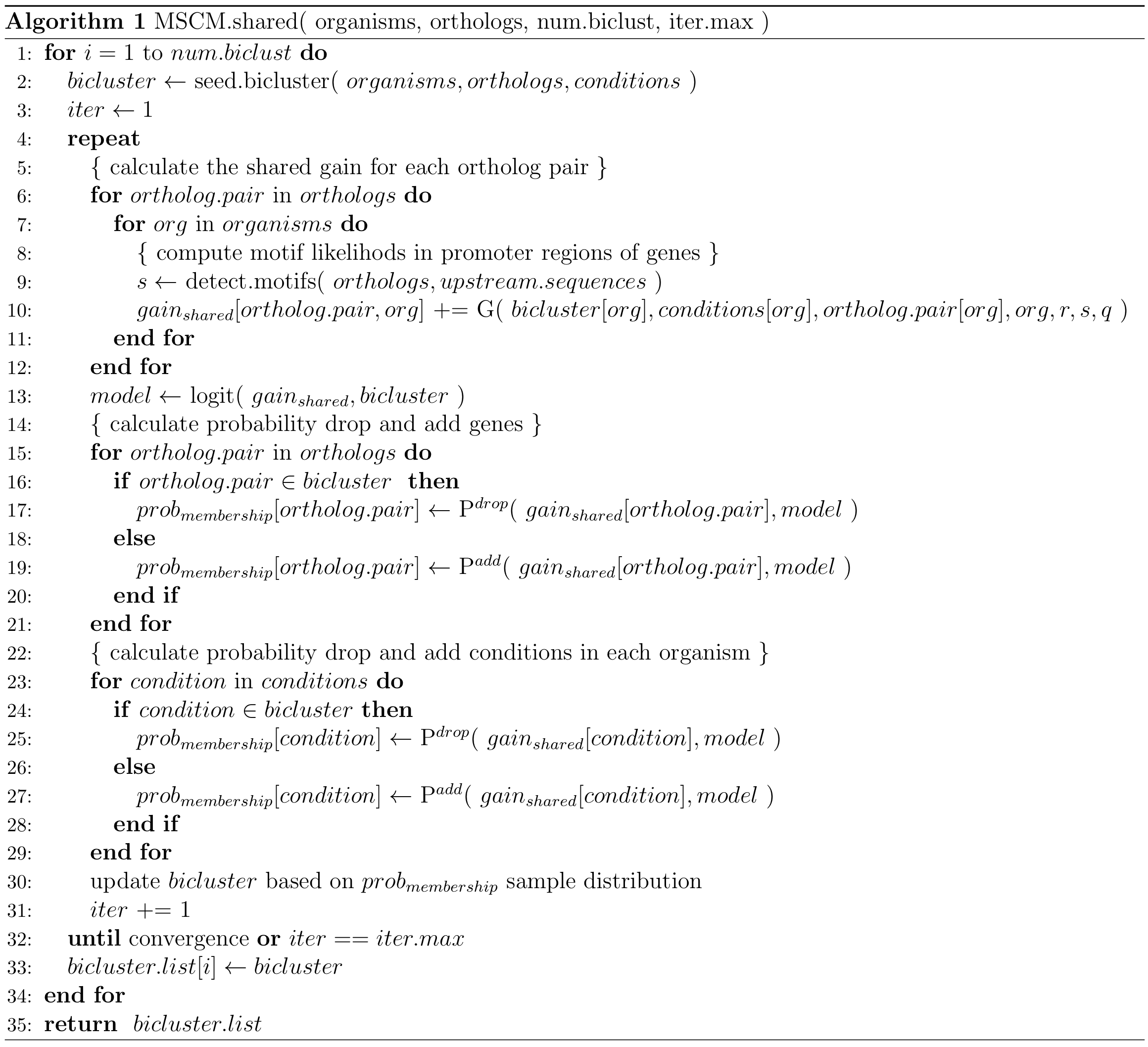
**

# Validation

**Table 3**: Quick lookup table for methods considered by this study.

|  | | Expression Only | |  | Full Data | |
| --- | --- | --- | --- | --- | --- | --- |
| Multi-Species | | Shared  space | full genome  (elaboration) |  | Shared  space | full genome  (elaboration) |
|  | cMonkey | EO-MSCM-SH | EO-MSCM-EL |  | FD-MSCM-SH | FD-MSCM-EL |
|  | ISA* | MSISA-P | MSISA-R |  | NA | NA |
|  | K-Means* | MSKM-SH | MSKM-EL |  | NA | NA |
|  | (Balanced) K-Means* | BMSKM-SH | BMSKM-EL |  | NA | NA |
|  | | | | | | |
| Single-Species | | Expression Only | |  | Full Data | |
|  | cMonkey | EO-SSCM | |  | FD-SSCM | |
|  | Coalesce | EO-COAL | |  | FD-COAL | |
|  | Qubic* | QUBIC | |  | NA | |
|  |  |  |  |  |  |  |
|  | * Expression only method by method definition - no distinction between "expression only" or "full data" is necessary. | | | | | |
|  |  |  |  |  |  |  |
|  |  |  |  |  |  |  |

## Overview of the bicluster comparison metrics

A comparison of the relative performances of four multi-species methods (MSCM, MSISA, MSKM and BMSKM), and three single species methods (SSCM, Coalesce and Qubic) in this study are based on 5 metric classes: 1) bicluster coherence; 2) functional enrichment; 3) coverage; 4) overlap between biclusters; and 5) conservation. Bicluster coherence is determined by the combination of five commonly used metrics that gauge the degree of support provided to each bicluster by the three data types that MScM integrates (expression, sequence and association networks). See [10] for comparisons of SSCM to other biclustering algorithms, and [19] comparisons between single species biclustering and clustering algorithms. Our coherence metrics are: 1) expression residuals – a measure of the coherence of expression across the two species datasets for conditions within the bicluster; 2) mean correlation – the average pairwise correlation between members of a (bi)cluster (taking the absolute value to allow fair comparison between methods that identify inversely correlated patterns (QUBIC and MSISA) and those that do not; 3) network p-values – a measure of the significance of the sub-networks within biclusters compared to the full network; 4) motif E-values – a measure of the quality/significance of the upstream binding site motifs detected for each (bi)cluster; and 5) sequence p-values – an estimate of a sequence’s match to the motifs associated with a (bi)cluster. Each of the coherence metrics is described in greater detail in [12].

## Quick-glance table for pairings involving *E. coli* – *S.* Typhimurium

**Table 4**: Summary of evaluation criteria for the single- and multi-species methods of the *E. coli* – *S.* Typhimurium pairing. We compare several metrics of bicluster conservation, coverage, and functional enrichment. In all cases metrics are averaged over all biclusters produced by that method. Abbreviations are given for each method; translations can be found in Table 3. In each column, the results for *E. coli* are listed first, with those for *S.* Typhimurium listed in parentheses.

| **Table 4** | | | | | | | | | GO | | | KEGG | |
| --- | --- | --- | --- | --- | --- | --- | --- | --- | --- | --- | --- | --- | --- |
|  | Conservation Score | Mean Correlation (absolute value) | Mean Net  p-value  (-log10) | Mean Number of Genes | Mean Number of Conditions | Number of Biclusters | Coverage (element wise) | Mean Overlap (element wise) | Percent (bi)clusters enriched  (pval < 0.01) | Number of Unique Enriched Terms | Percent (bi)clusters enriched  (pval < 0.01) | | Number of Unique Enriched Pathways |
| *E. coli* | | | | | | | | | | | | | |
| EO MSCM-SH | 1 | 0.52 | 7.51 | 20.95 | 230.65 | 150 | 0.25% | 0.04% | 33.33% | 479 | 9.33% | | 19 |
| FD MSCM-SH | 1 | 0.68 | 16.4 | 26.28 | 227.58 | 149 | 0.26% | 0.06% | 65.10% | 806 | 23.49% | | 33 |
| ISA-P | 1 | 0.56 | 3.78 | 7.78 | 25.72 | 60 | 0.00% | 0.10% | 45.00% | 228 | 23.33% | | 17 |
| MSKM-SH | 1 | 0.59 | 9.64 | 19.07 | 507 | 148 | 0.66% | 0.00% | 62.84% | 918 | 15.54% | | 33 |
| BMSKM-SH | 1 | 0.54 | 11.77 | 18.85 | 507 | 150 | 0.66% | 0.00% | 58.00% | 885 | 12.00% | | 32 |
| EO MSCM-EL | 0.894 | 0.54 | 4.71 | 29.13 | 231.82 | 150 | 0.31% | 0.04% | 46.67% | 617 | 11.33% | | 25 |
| FD MSCM-EL | 0.764 | 0.66 | 19.92 | 39.65 | 227.23 | 149 | 0.34% | 0.06% | 89.93% | 999 | 48.32% | | 58 |
| ISA-R | 0.022 | 0.52 | 6.13 | 38.85 | 25.72 | 60 | 0.02% | 0.06% | 90.00% | 570 | 31.67% | | 37 |
| MSKM-EL | 0.994 | 0.57 | 8.84 | 28.81 | 507 | 148 | 1.00% | 0.00% | 69.59% | 1037 | 16.22% | | 40 |
| BMSKM-EL | 0.995 | 0.54 | 9.61 | 28.43 | 507 | 150 | 1.00% | 0.00% | 71.33% | 1054 | 13.33% | | 32 |
| EO SSCM | 0.106 | 0.76 | 6.73 | 26.31 | 346.84 | 204 | 0.46% | 0.13% | 59.80% | 926 | 17.16% | | 44 |
| FD SSCM | 0.1 | 0.59 | 19.5 | 19.4 | 354.48 | 425 | 0.69% | 0.11% | 64.24% | 1221 | 12.71% | | 47 |
| EO COAL | 0.097 | 0.64 | 6.28 | 70.53 | 39.71 | 239 | 0.25% | 0.03% | 77.41% | 986 | 33.47% | | 49 |
| FD COAL | 0.095 | 0.63 | 6.18 | 70.43 | 38.96 | 247 | 0.26% | 0.03% | 79.76% | 984 | 35.22% | | 48 |
| QUBIC | 0.038 | 0.91 | 27.73 | 6.67 | 27.45 | 139 | 0.01% | 0.24% | 76.26% | 437 | 38.85% | | 21 |
| *S.* Typhimurium | | | | | | | | | | | | | |
| EO MSCM-SH | - | 0.45 | 3.56 | 20.95 | 58.93 | 150 | 0.26% | 0.04% | 36.67% | 453 | 10.67% | | 18 |
| FD MSCM-SH | - | 0.55 | 13.65 | 26.28 | 56.08 | 149 | 0.27% | 0.07% | 67.11% | 656 | 21.48% | | 38 |
| ISA-P | - | 0.6 | 8.43 | 7.78 | 11.88 | 60 | 0.01% | 0.26% | 33.33% | 175 | 20.00% | | 12 |
| MSKM-SH | - | 0.29 | 5.65 | 19.07 | 138 | 148 | 0.75% | 0.00% | 61.49% | 742 | 18.24% | | 39 |
| BMSKM-SH | - | 0.37 | 4.4 | 18.85 | 138 | 150 | 0.75% | 0.00% | 54.67% | 739 | 12.00% | | 32 |
| EO MSCM-EL | - | 0.47 | 3.35 | 27.72 | 62.19 | 150 | 0.32% | 0.04% | 35.33% | 424 | 10.00% | | 19 |
| FD MSCM-EL | - | 0.5 | 16.81 | 36.64 | 56.15 | 149 | 0.33% | 0.05% | 81.21% | 720 | 40.94% | | 53 |
| ISA-R | - | 0.46 | 3.97 | 189.47 | 13.5 | 60 | 0.03% | 0.91% | 18.33% | 63 | 5.00% | | 2 |
| MSKM-EL | - | 0.31 | 4.98 | 25.3 | 138 | 148 | 1.00% | 0.00% | 58.78% | 721 | 18.24% | | 37 |
| BMSKM-EL | - | 0.38 | 3.87 | 24.97 | 138 | 150 | 1.00% | 0.00% | 51.33% | 728 | 12.00% | | 32 |
| EO SSCM | - | 0.66 | 3.58 | 27.54 | 91.96 | 155 | 0.41% | 0.11% | 29.03% | 355 | 5.16% | | 12 |
| FD SSCM | - | 0.58 | 5 | 29.97 | 94.13 | 157 | 0.38% | 0.14% | 28.66% | 316 | 5.73% | | 9 |
| EO COAL | - | 0.57 | 3.18 | 100.58 | 14.89 | 159 | 0.51% | 0.02% | 32.08% | 388 | 3.77% | | 14 |
| FD COAL | - | 0.57 | 3.16 | 100.67 | 14.88 | 159 | 0.51% | 0.02% | 33.33% | 391 | 5.03% | | 11 |
| QUBIC | - | 0.86 | 6.33 | 6.88 | 5.41 | 113 | 0.01% | 0.08% | 14.16% | 84 | 3.54% | | 2 |

## Additional (bi)cluster coherence metric figures

### Residuals

**Figure 5**: Boxplot of residuals from the *E. coli* – *S.* Typhimurium pairing. The distributions of the residuals from all methods considered by this study for the *E. coli*- *S.* Typhimurium pairing. Explanations of the method name abbreviations can be found in Table 3.


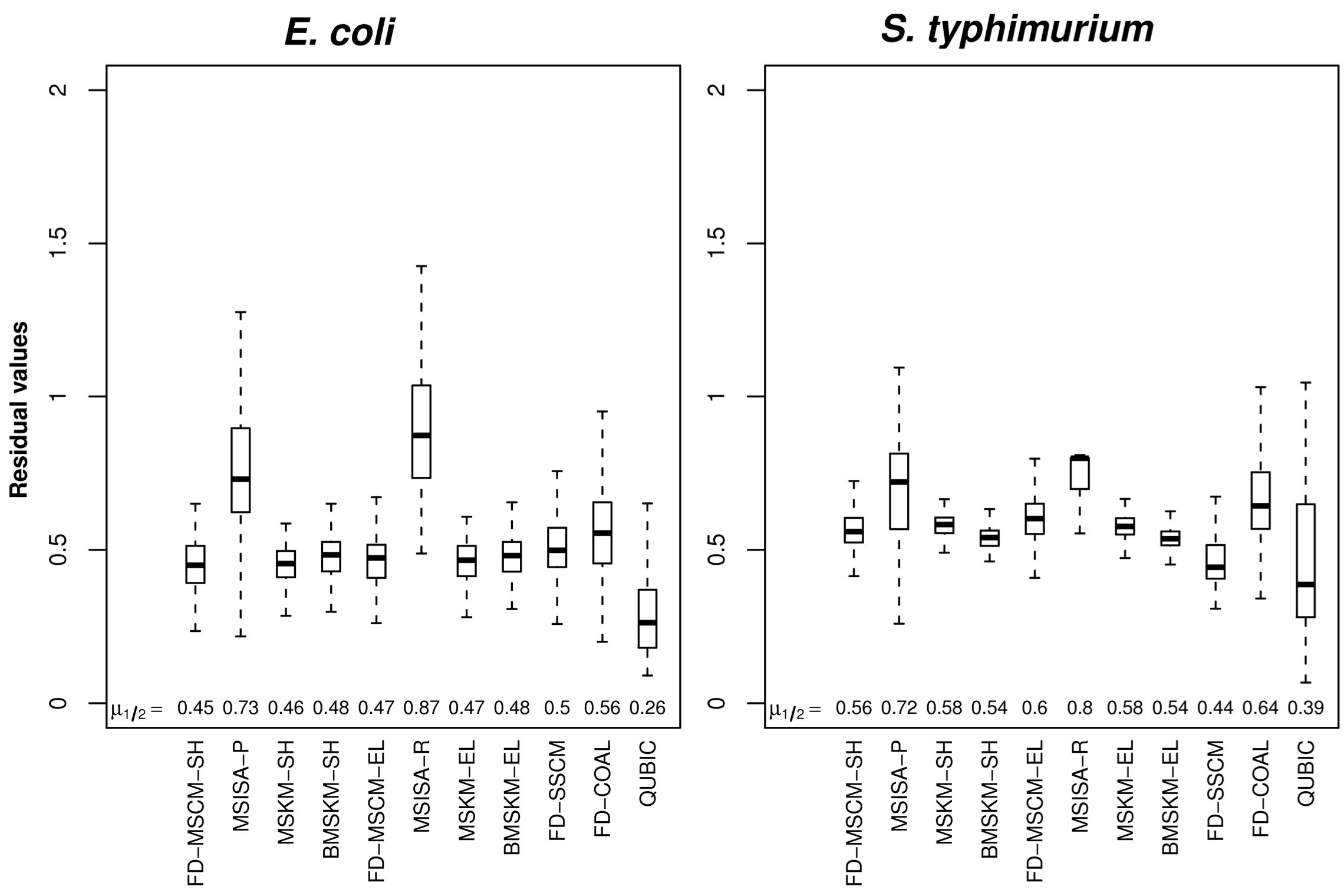


*E. coli*

*S.* Typhimurium

### Average pairwise correlations

**Figure 6**: Mean correlations from the *E. coli* – *S.* Typhimurium pairing. The distributions of the mean correlations from all methods considered by this study for the *E. coli*- *S.* Typhimurium pairing. Explanations of the method name abbreviations can be found in Table 3.

*E. coli*

*S.* Typhimurium


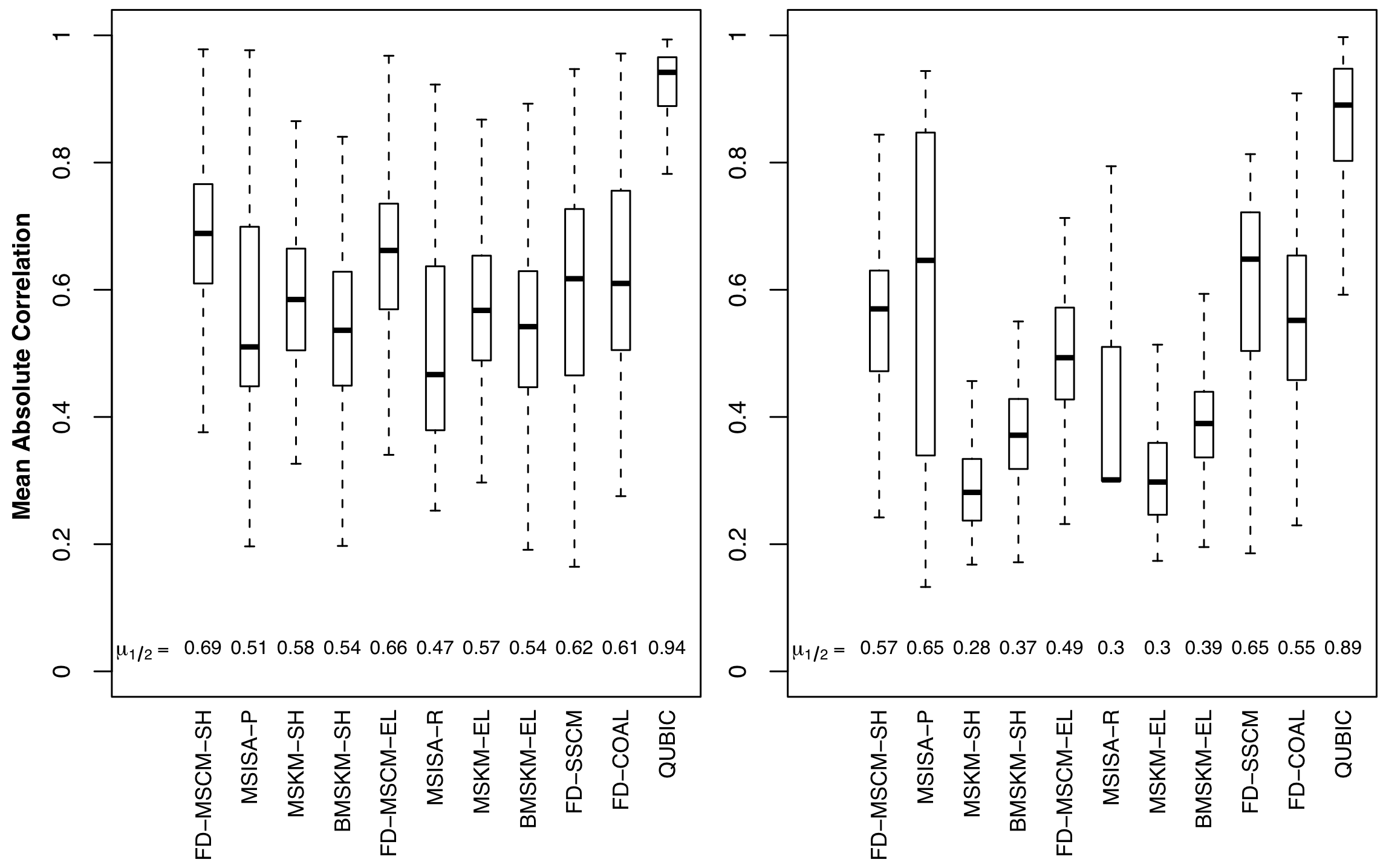


### Network Association p-values

**Figure 7**: Network Association p-values from the *E. coli* – *S.* Typhimurium pairing. The distributions of the network association p-values (-log10) from all methods considered by this study for the *E. coli*- *S.* Typhimurium pairing. Explanations of the method name abbreviations can be found in Table 3.

*E. coli*

*S.* Typhimurium

##
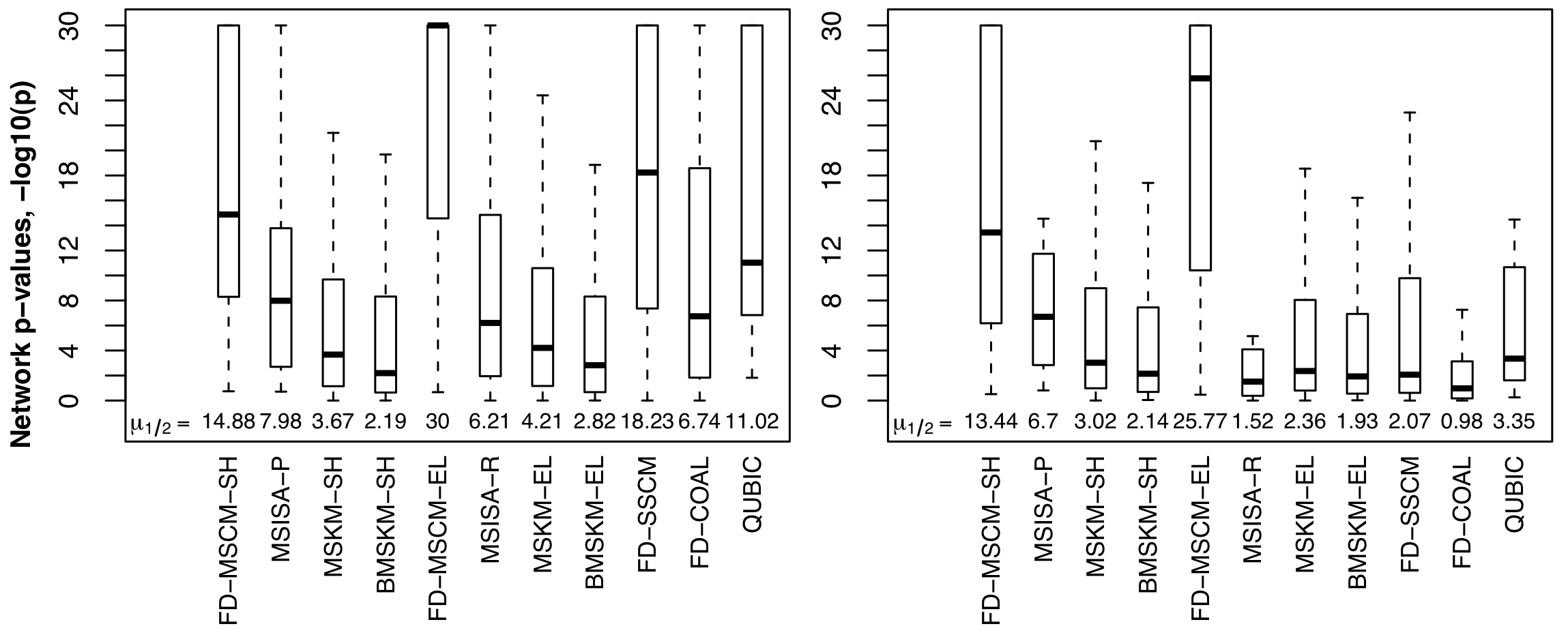


## Additional size distribution, overlap and coverage figures

### Number of genes

**Figure 8**: Number of genes from the *E. coli* – *S.* Typhimurium pairing. The distributions of the number of genes from all methods considered by this study for the *E. coli* – *S.* Typhimurium pairing. Explanations of the method name abbreviations can be found in Table 3.

*E. coli*

*S.* Typhimurium


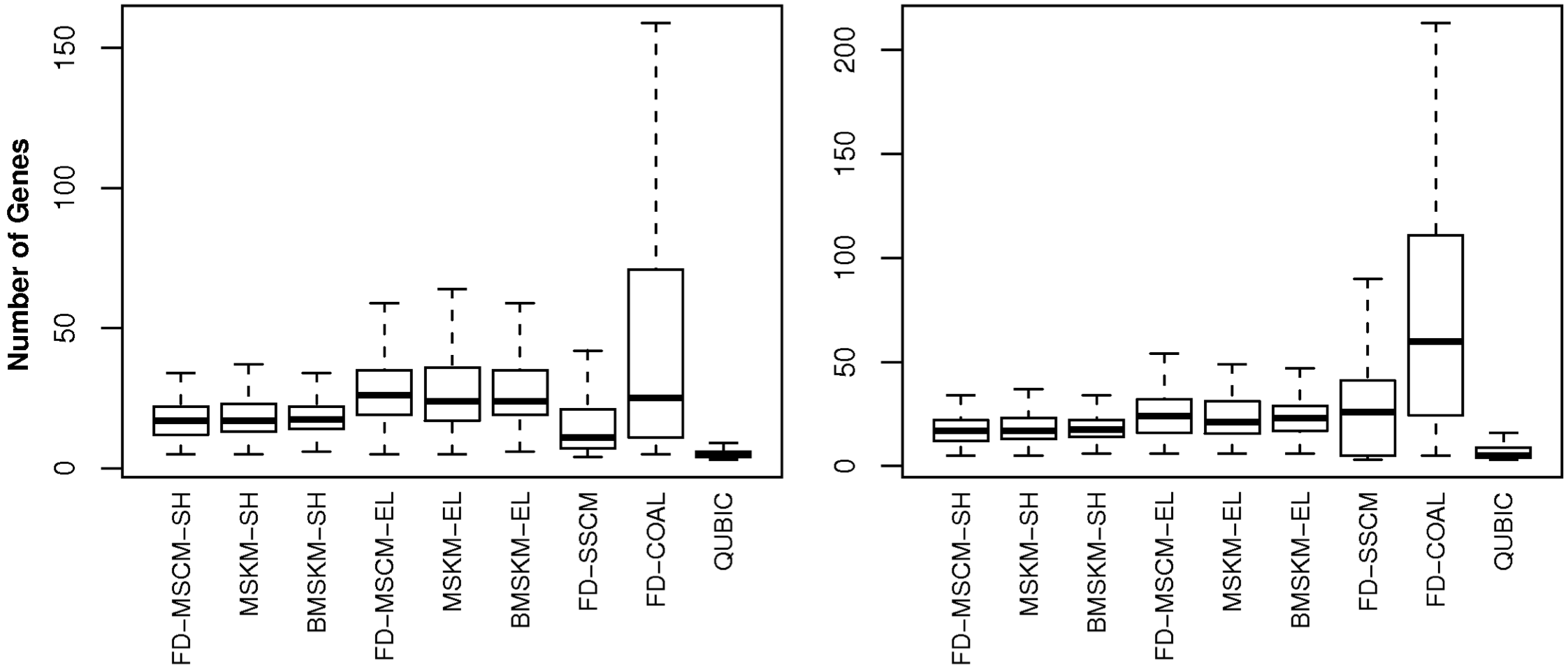


### Number of conditions

**Figure 9**: Number of conditions from the *E. coli* – *S.* Typhimurium pairing. The distributions of the number of conditions from all methods considered by this study for the *E. coli* – *S.* Typhimurium pairing. Explanations of the method name abbreviations can be found in Table 3.

*E. coli*

*S.* Typhimurium


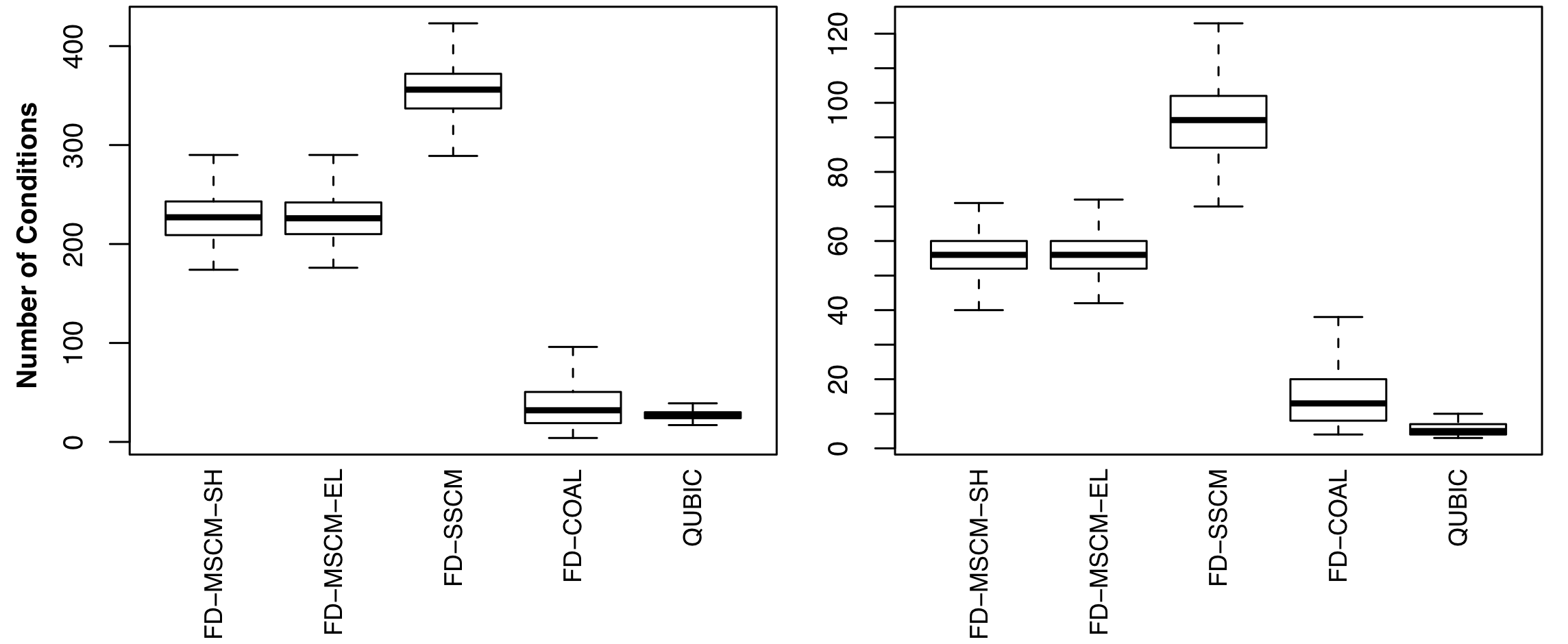


### Coverage (matrix element-wise)

**Figure 10**: Coverages (matrix element-wise) from the *E. coli* – *S.* Typhimurium pairing. The distributions of the Coverages (matrix element-wise) from all methods considered by this study for the *E. coli* – *S.* Typhimurium pairing. Explanations of the method name abbreviations can be found in Table 3.

*E. coli*

*S.* Typhimurium


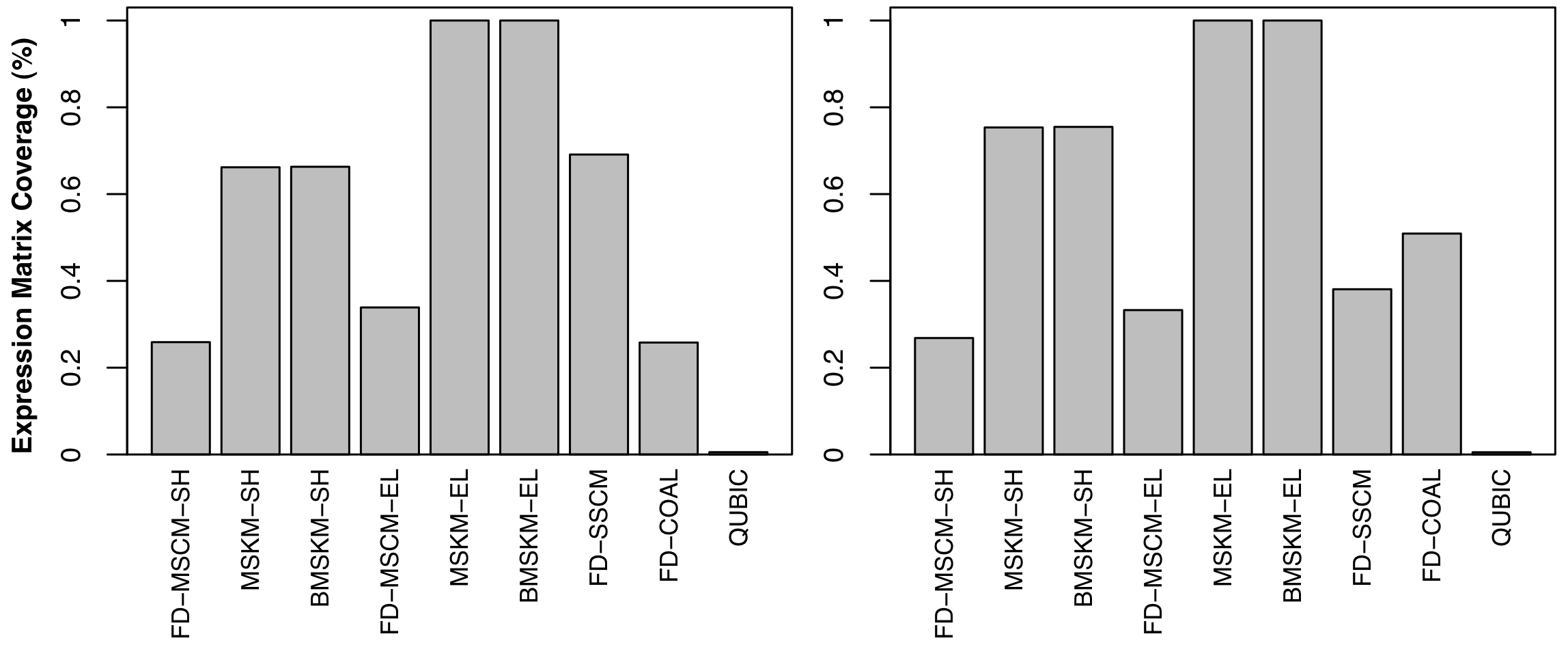


### Overlap (matrix element-wise)

**Figure 11**: Overlaps (matrix element-wise) from the *E. coli* – *S.* Typhimurium pairing. The distributions of the Overlaps (matrix element-wise) from all methods considered by this study for the *E. coli* – *S.* Typhimurium pairing. Explanations of the method name abbreviations can be found in Table 3.

*E. coli*

*S.* Typhimurium


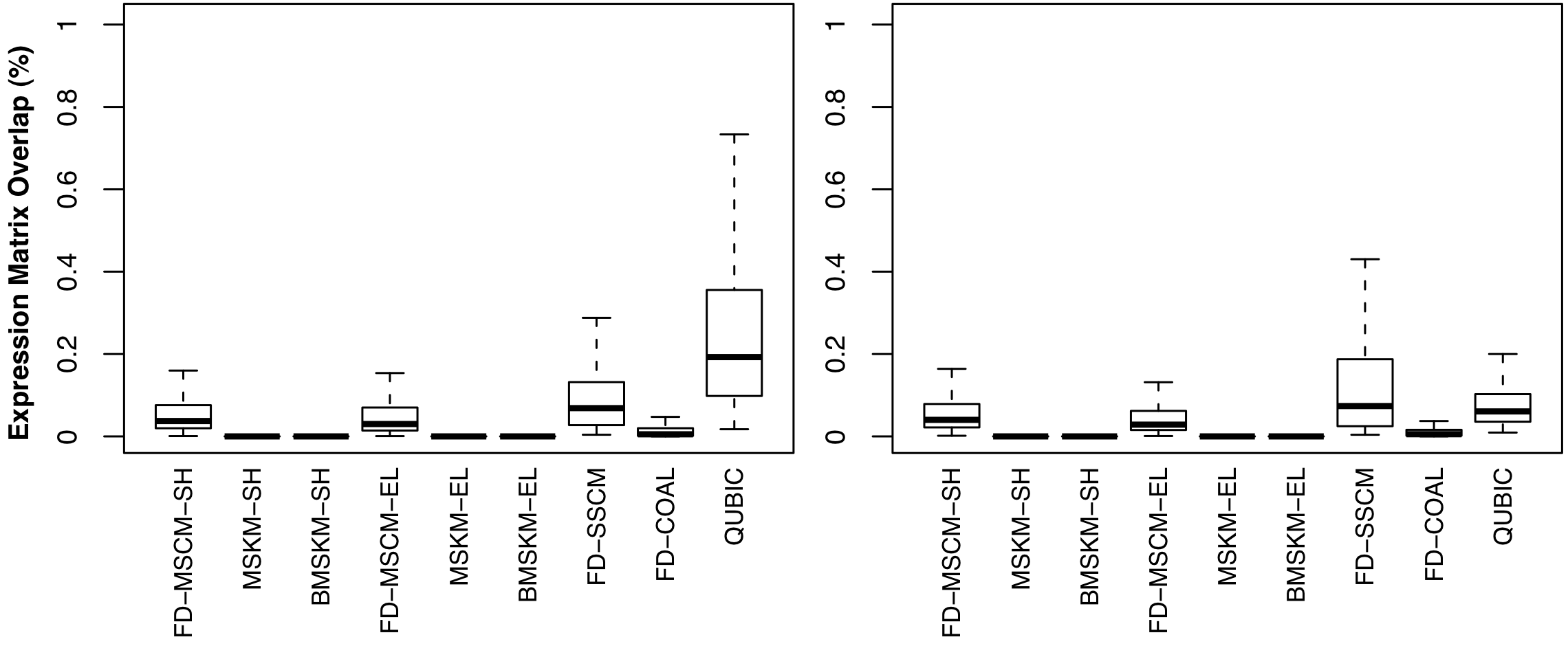


### GO and KEGG annotation enrichment

**Figure 12**: Comparison of the fraction of biclusters with significant GO and KEGG annotation enrichments from all methods considered by this study for the *E. coli* – *S.* Typhimurium pairing. (A) Percentage of biclusters with enriched GO terms. (B) Percentage of biclusters with enriched KEGG pathways. Explanations of the method name abbreviations can be found in Table 3.


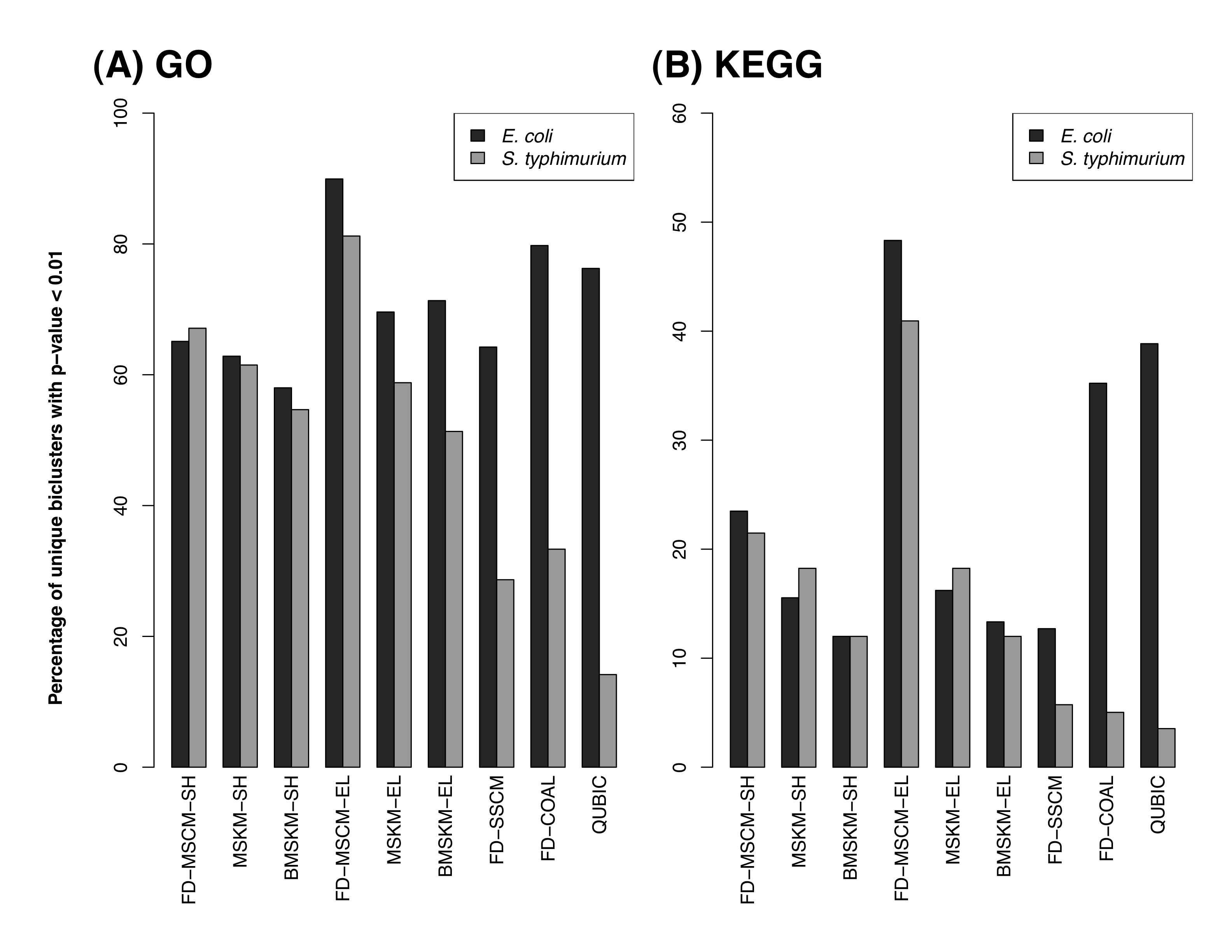


# Description of highlighted biclusters

## *E. coli* bicluster 57

**Figure 13**: *E. coli* bicluster 57 MScM output image


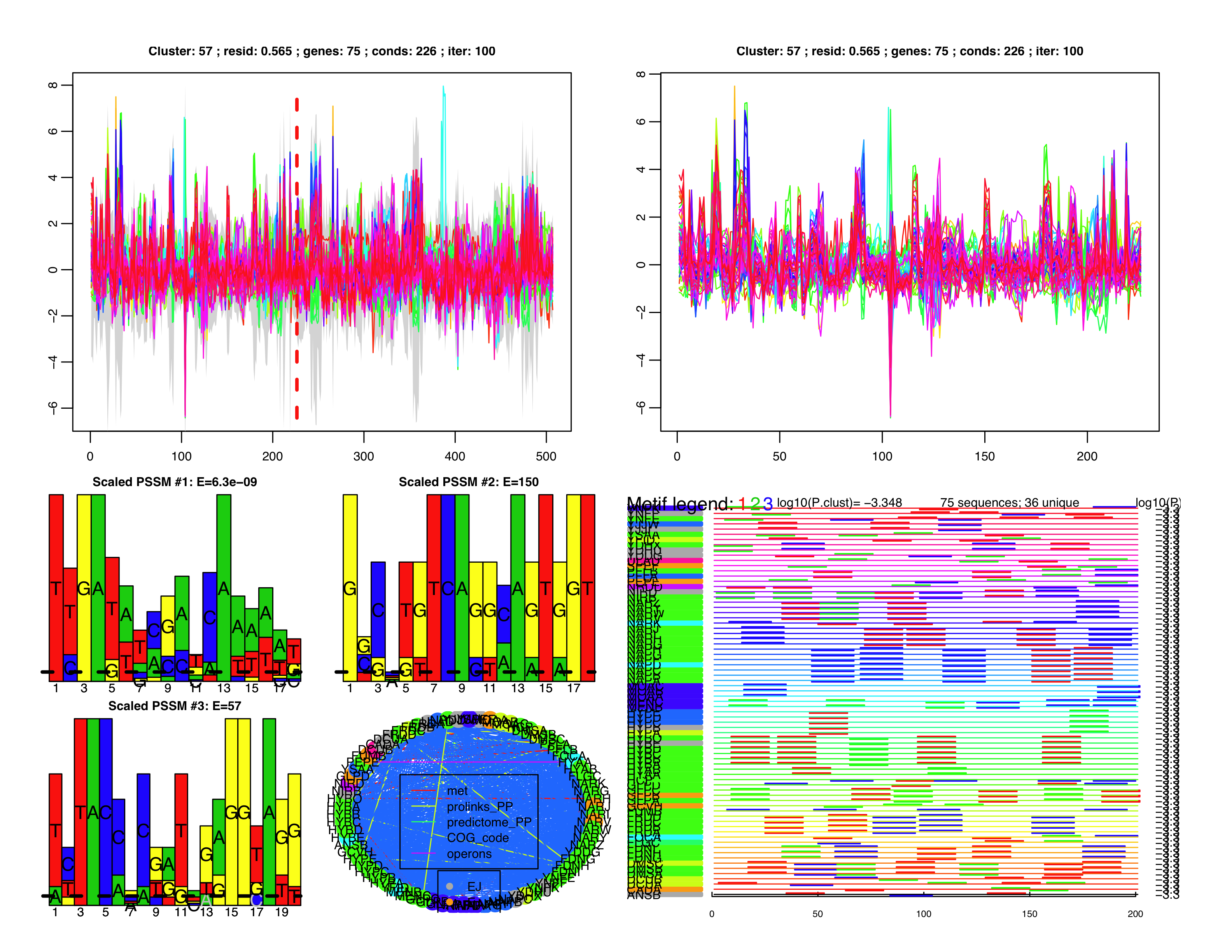


### *E. coli* bicluster 57 core gene list

| **Locus** | **Name** | **description** |
| --- | --- | --- |
| B0693 | SPEF | ornithine decarboxylase isozyme, inducible |
| B0782 | MOAB | molybdopterin biosynthesis protein B |
| B0783 | MOAC | molybdopterin biosynthesis, protein C |
| B0873 | HCP | hybrid-cluster [4Fe-2S-2O] protein in anaerobic |
| B0894 | DMSA | dimethyl sulfoxide reductase, anaerobic, subunit |
| B0895 | DMSB | dimethyl sulfoxide reductase, anaerobic, subunit |
| B0896 | DMSC | dimethyl sulfoxide reductase, anaerobic, subunit |
| B0903 | PFLB | pyruvate formate lyase I |
| B1074 | FLGC | flagellar component of cell-proximal portion of |
| B1587 | YNFE | oxidoreductase subunit |
| B1588 | YNFF | oxidoreductase subunit |
| B1476 | FDNI | formate dehydrogenase-N, cytochrome B556 (gamma) |
| B1475 | FDNH | formate dehydrogenase-N, Fe-S (beta) subunit, nitrate-inducible |
| B1474 | FDNG | formate dehydrogenase-N, alpha subunit, nitrate-inducible |
| B1227 | NARI | nitrate reductase 1, gamma (cytochrome b(NR)) |
| B1226 | NARJ | molybdenum-cofactor-assembly chaperone subunit |
| B1225 | NARH | nitrate reductase 1, beta (Fe-S) subunit |
| B1224 | NARG | nitrate reductase 1, alpha subunit |
| B1223 | NARK | nitrate/nitrite transporter |
| B2202 | NAPC | nitrate reductase, cytochrome c-type, periplasmic |
| B2203 | NAPB | nitrate reductase, small, cytochrome C550 |
| B2204 | NAPH | ferredoxin-type protein essential for electron |
| B2205 | NAPG | ferredoxin-type protein essential for electron |
| B2206 | NAPA | nitrate reductase, periplasmic, large subunit |
| B2207 | NAPD | assembly protein for periplasmic nitrate |
| B2208 | NAPF | ferredoxin-type protein, predicted role in |
| B2261 | MENC | o-succinylbenzoyl-CoA synthase |
| B2262 | MENB | dihydroxynaphthoic acid synthetase |
| B2997 | HYBO | hydrogenase 2, small subunit |
| B4131 | CADA | lysine decarboxylase 1 |
| B2727 | HYPB | GTP hydrolase involved in nickel liganding into |
| B2728 | HYPC | protein required for maturation of hydrogenases |
| B2729 | HYPD | protein required for maturation of hydrogenases |
| B2904 | GCVH | glycine cleavage complex lipoylprotein |
| B2957 | ANSB | periplasmic L-asparaginase II |
| B2992 | HYBE | hydrogenase 2-specific chaperone |
| B2993 | HYBD | predicted maturation element for hydrogenase 2 |
| B2995 | HYBB | predicted hydrogenase 2 cytochrome b type |
| B2996 | HYBA | hydrogenase 2 4Fe-4S ferredoxin-type component |
| B3573 | YSAA | predicted hydrogenase, 4Fe-4S ferredoxin-type |
| B4021 | PEPE | (alpha)-aspartyl dipeptidase |
| B4122 | FUMB | anaerobic class I fumarate hydratase (fumarase |
| B4123 | DCUB | C4-dicarboxylate antiporter |
| B4138 | DCUA | C4-dicarboxylate antiporter |
| B4151 | FRDD | fumarate reductase (anaerobic), membrane anchor |
| B4152 | FRDC | fumarate reductase (anaerobic), membrane anchor |
| B4153 | FRDB | fumarate reductase (anaerobic), Fe-S subunit |
| B4154 | FRDA | fumarate reductase (anaerobic) catalytic and |
| B4196 | ULAD | 3-keto-L-gulonate 6-phosphate decarboxylase |
| B4238 | NRDD | anaerobic ribonucleoside-triphosphate reductase |
| B4380 | YJJI | conserved protein |

### *E. coli* bicluster 57 elaborated gene list

| **Locus** | **Name** | **Description** |
| --- | --- | --- |
| B0781 | MOAA | molybdopterin biosynthesis protein A |
| B0902 | PFLA | pyruvate formate lyase activating enzyme 1 |
| B0904 | FOCA | formate channel |
| B0972 | HYAA | hydrogenase 1, small subunit |
| B0973 | HYAB | hydrogenase 1, large subunit |
| B1465 | NARV | nitrate reductase 2 (NRZ), gamma subunit |
| B1466 | NARW | nitrate reductase 2 (NRZ), delta subunit |
| B1467 | NARY | nitrate reductase 2 (NRZ), beta subunit |
| B1468 | NARZ | nitrate reductase 2 (NRZ), alpha subunit |
| B1473 | YDDG | aromatic amino acid exporter |
| B1593 | YNFK | predicted dethiobiotin synthetase |
| B1670 | YDHU | predicted cytochrome |
| B1671 | YDHX | predicted 4Fe-4S ferridoxin-type protein |
| B2241 | GLPA | sn-glycerol-3-phosphate dehydrogenase |
| B2242 | GLPB | sn-glycerol-3-phosphate dehydrogenase |
| B2243 | GLPC | sn-glycerol-3-phosphate dehydrogenase |
| B2579 | YFID | autonomous glycyl radical cofactor |
| B2726 | HYPA | protein involved in nickel insertion into |
| B2730 | HYPE | carbamoyl dehydratase, hydrogenases 1,2,3 |
| B2994 | HYBC | hydrogenase 2, large subunit |
| B3365 | NIRB | nitrite reductase, large subunit, NAD(P)H-binding |
| B3366 | NIRD | nitrite reductase, NAD(P)H-binding, small |
| B3426 | GLPD | sn-glycerol-3-phosphate dehydrogenase, aerobic, FAD/NAD(P)-binding |
| B4379 | YJJW | predicted pyruvate formate lyase activating |

## *S.* Typhimurium bicluster 57

**Figure 14**: *S.* Typhimurium bicluster 57 MScM output image


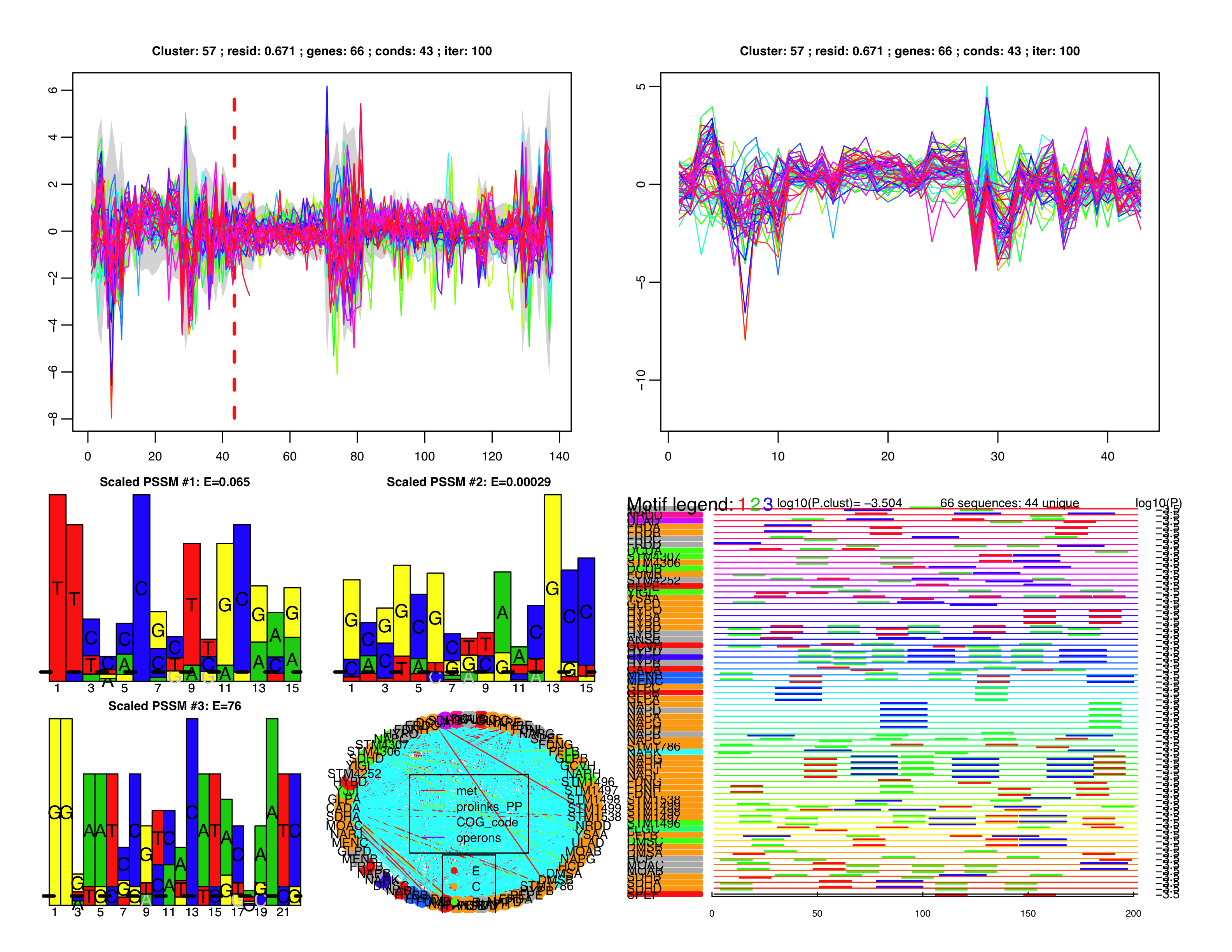


### *S.* Typhimurium bicluster 57 core gene list

| Locus | Name | description |
| --- | --- | --- |
| STM0701 | SPEF | ornithine decarboxylase isozyme |
| STM0803 | MOAB | molybdopterin biosynthetic protein B |
| STM0804 | MOAC | molybdenum cofactor biosynthesis protein C |
| STM0937 | HCP | hydroxylamine reductase |
| STM0964 | DMSA | anaerobic dimethyl sulfoxide reductase subunit |
| STM0965 | DMSB | anaerobic dimethyl sulfoxide reductase subunit |
| STM0966 | DMSC | anaerobic dimethyl sulfoxide reductase subunit |
| STM0973 | PFLB | pyruvate formate lyase I |
| STM1175 | FLGC | flagellar basal body rod protein FlgC |
| STM1498 |  | putative dimethyl sulphoxide reductase |
| STM1499 |  | putative dimethyl sulphoxide reductase chain A1 |
| STM1568 | FDNI | formate dehydrogenase-N subunit gamma |
| STM1569 | FDNH | formate dehydrogenase-N beta subunit |
| STM1570 | FDNG | formate dehydorgenase-N alpha subunit |
| STM1761 | NARI | nitrate reductase 1 gamma subunit |
| STM1762 | NARJ | nitrate reductase 1 delta subunit |
| STM1763 | NARH | nitrate reductase 1 beta subunit |
| STM1764 | NARG | nitrate reductase 1 alpha subunit |
| STM1765 | NARK | nitrite extrusion protein |
| STM2255 | NAPC | cytochrome c-type protein NapC |
| STM2256 | NAPB | diheme cytochrome c550 |
| STM2257 | NAPH | quinol dehydrogenase membrane component |
| STM2258 | NAPG | quinol dehydrogenase periplasmic component |
| STM2259 | NAPA | periplasmic nitrate reductase |
| STM2260 | NAPD | assembly protein for periplasmic nitrate |
| STM2261 | NAPF | ferredoxin-type protein |
| STM2306 | MENC | O-succinylbenzoate synthase |
| STM2307 | MENB | naphthoate synthase |
| STM3150 | HYPO | hydrogenase 2 small subunit |
| STM2559 | CADA | lysine decarboxylase 1 |
| STM2855 | HYPB | hydrogenase nickel incorporation protein HypB |
| STM2856 | HYPC | hydrogenase isoenzymes formation protein |
| STM2857 | HYPD | putative hydrogenase formation protein |
| STM3054 | GCVH | glycine cleavage system protein H |
| STM3106 | ANSB | L-asparaginase II |
| STM3145 | HYBE | hydrogenase 2-specific chaperone |
| STM3146 | HYBD | predicted maturation element for hydrogenase 2 |
| STM3148 | HYBB | predicted hydrogenase 2 cytochrome b type |
| STM3149 | HYBA | hydrogenase 2 protein HybA |
| STM3666 | YSAA | putative oxidoreductase |
| STM4190 | PEPE | peptidase E |
| STM4300 | FUMB | fumarase B |
| STM4301 | DCUB | anaerobic C4-dicarboxylate transporter |
| STM4325 | DCUA | anaerobic C4-dicarboxylate transporter |
| STM4340 | FRDD | fumarate reductase subunit D |
| STM4341 | FRDC | fumarate reductase subunit C |
| STM4342 | FRDB | fumarate reductase iron-sulfur subunit |
| STM4343 | FRDA | fumarate reductase flavoprotein subunit |
| STM4386 | ULAD | 3-keto-L-gulonate-6-phosphate decarboxylase |
| STM4452 | NRDD | anaerobic ribonucleoside triphosphate reductase |
| STM4566 | YJJI | hypothetical protein |

### *S.* Typhimurium bicluster 57 elaborated gene list

| Locus | Name | description |
| --- | --- | --- |
| STM0733 | SDHD | succinate dehydrogenase cytochrome b556 small |
| STM0734 | SDHA | succinate dehydrogenase flavoprotein subunit |
| STM0735 | SDHB | succinate dehydrogenase iron-sulfur subunit |
| STM1496 | STM1496 | putative dimethylsulfoxide reductase |
| STM1497 | STM1497 | putative dimethyl sulphoxide reductase |
| STM1538 | STM1538 | putative hydrogenase-1 large subunit |
| STM1786 | STM1786 | hydrogenase-1 small subunit |
| STM2284 | GLPA | sn-glycerol-3-phosphate dehydrogenase subunit A |
| STM2285 | GLPB | anaerobic glycerol-3-phosphate dehydrogenase |
| STM2286 | GLPC | sn-glycerol-3-phosphate dehydrogenase subunit C |
| STM3526 | GLPD | glycerol-3-phosphate dehydrogenase |
| STM3962 | YIGL | predicted hydrolase |
| STM4252 | STM4252 | putative inner membrane protein |
| STM4306 | STM4306 | putative anaerobic dimethylsulfoxide reductase |
| STM4307 | STM4307 | putative anaerobic dimethylsulfoxide reductase |
